# Urgent considerations on renal immune-related adverse events in oncology practice

**DOI:** 10.3389/fimmu.2025.1714090

**Published:** 2026-02-10

**Authors:** Wen-Qing Lv, Jing-Yao Lu, Jun Li, Dao-Yuan Lv, Qi Ke

**Affiliations:** 1 Department of Nephrology, Affiliated Hospital of Jiangnan University, Wuxi, Jiangsu, China; 2 Wuxi School of Medicine, Jiangnan University, Wuxi, Jiangsu, China

**Keywords:** immune checkpoint inhibitors, renal immune-related adverse events, chronic kidney disease, mechanism, biomarkers, treatment

## Abstract

In recent years, immune checkpoint inhibitors (ICIs) have emerged as a critical component of hematological malignancies and solid malignant tumors therapy. However, clinical practice has revealed that these agents may induce immune-related adverse events (irAEs). Notably, renal irAEs stands out as a significant clinical concern, frequently necessitating treatment discontinuation and thereby enabling tumor progression. Renal irAEs constitutes a critical consideration for patients with cancer complicated by chronic kidney disease (CKD). This review systematically examines the immunologic pathogenesis of ICIs-induced renal disease, susceptibility genes, non-invasive biomarkers, and efficient intervention strategies. It further analyzes the critical considerations regarding renal irAEs that oncologists must address, based on real-world evidence from ICIs therapy in cancer patients with CKD, including those who are renal transplantation recipients or have end-stage renal disease (ESRD). Additionally, the promising targeted immunotherapy for malignant tumors is expected to improve both renal outcomes and survival prognosis in cancer patients.

## 1 Introduction

In recent years, immune checkpoint inhibitors (ICIs) have been widely used in the treatment of patients with metastatic renal cell carcinoma, hepatocellular carcinoma, non-small cell lung cancer, breast cancer, Hodgkin lymphoma, advanced melanoma, head and neck squamous cell carcinoma, as well as other malignancies, contributing to prolonged survival and improved life expectancy in this population (1). However, nephrotoxicity remains a significant safety concern associated with the administration of ICIs.

On one hand, immune checkpoint inhibitor-related acute kidney injury (ICIs-AKI) may impair the tolerance to anti-tumor therapies. Although ICIs are not renally metabolized (2), many other chemotherapeutic agents rely on renal excretion. On the other hand, kidney injury can lead to renal anemia, disturbances in fluid, electrolyte, and acid-base homeostasis, impaired immune function, and organ dysfunction due to accumulation of uremic toxins, thereby exacerbating the adverse effects of anticancer treatments. Therefore, early diagnosis and timely intervention for ICIs-AKI are of critical importance for improving renal outcomes and survival prognosis in cancer patients.

Currently, the identification and diagnosis of ICIs-AKI primarily rely on renal biopsy; however, the use of non-invasive biomarkers holds promise for early intervention and may contribute to improved renal outcomes. The non-invasive biomarkers may also reduce the necessity for renal biopsies, thus promoting faster patient recovery. Furthermore, ICIs-AKI may contribute to organ dysfunction and reduce survival rates in patients with malignant tumors, highlighting the critical need to identify susceptibility genes for early prediction.

Reduced estimated glomerular filtration rate (eGFR) has been significantly associated with increased risk of incident cancers in specific sites, including the renal tract (encompassing kidney, ureter, and bladder), oropharynx, respiratory system, hematologic tissues (such as myeloma and leukemia), skin, and abdominal solid organs (3, 4). In a cohort of 13,750 individuals diagnosed with concomitant chronic kidney disease (CKD), 2,758 (20.1%) subsequently developed cancer, with a median interval of 8.5 years between CKD diagnosis and cancer onset (5). In another study involving 431,263 participants from the UK Biobank—enrolled between 2007 and 2010 and without prior cancer history—41,745 new cancer cases and 11,764 cancer-related deaths were recorded over a median follow-up period of 11.3 years (6). Additionally, the 5-year cumulative incidence of any cancer among 482,510 incident hemodialysis patients was 9.48% (7). Up to 32% of cancer patients have CKD, and approximately two-thirds of this subgroup exhibit a glomerular filtration rate between 30 and 60 mL/min per 1.73 m², a range in which the selection of antineoplastic agents and dosage adjustments are typically required (8). Fortunately, the efficacy and safety of ICIs in patients with pre-existing renal dysfunction have been established (9, 10), which brings hope for cancer treatment in those with renal insufficiency. Given the adverse effects associated with ICIs administration, targeted delivery of ICIs may provide significant advantages by preserving renal function, maintaining survival rates, and improving quality of life during cancer therapy.

This review aims to provide a comprehensive overview of the epidemiological characteristics, pathogenesis, susceptibility genes, non-invasive biomarkers, therapeutic strategies, and prognosis associated with ICIs-AKI. Additionally, this article outlines the safety considerations of administering ICIs in cancer patients with CKD, including those who are renal transplantation recipients or have end-stage renal disease (ESRD), and reviews emerging targeted immunotherapeutic strategies designed to prevent renal irAEs.

## 2 Classification of ICIs and their anti-tumor mechanism

There are four types of ICIs currently approved for clinical use, as shown in **Table 1**.

**Table 1:** Classification of ICIs.

Classification	Active ingrediant	Drug name	Dosage form
CTLA-4 inhibitors	Ipilimumab	YERVOY	INJECTABLE; INJECTION
	Tremelimumab	IMJUDO	INJECTABLE; INTRAVENOUS
PD-1 inhibitors	Nivolumab	OPDIVO	INJECTABLE; INJECTION
	Pembrolizumab	KEYTRUDA	SOLUTION; INTRAVENOUS
	Toripalimab	LOQTORZI	INJECTABLE; INJECTION
	Cemiplimab	LIBTAYO	INJECTABLE; INTRAVENOUS
	Tislelizumab	TEVIMBRA	INJECTION; SOLUTION
PD-L1 inhibitors	Durvalumab	IMFINZI	INJECTABLE; INJECTION
	Atezolizumab	TECENTRIQ	INJECTABLE; INJECTION
	Avelumab	BAVENCIO	INJECTABLE; INJECTION; INTRAVENOUS
LAG3 inhibitors	Relatlimab	OPDUALAG	SOLUTION;INTRAVENOUS

All data is sourced from U.S. Food and Drug Administration.(https://www.fda.gov/).

CTLA4, cytotoxic T lymphocyte-associated antigen-4; PD-1, programmed cell death protein-1; LAG3,Lymphocyte-activation gene 3.

Cytotoxic T-lymphocyte-associated protein 4 (CTLA-4) is expressed on a variety of immune cells, including activated CD4^+^ and CD8^+^T cells, regulatory T cells, and tumor-infiltrating natural killer cells (11). Following T cell receptor activation and concurrent CD28-mediated co-stimulation, CTLA-4 is upregulated and translocated to the cell surface, where it competes with CD28 for binding to the critical co-stimulatory ligands CD80 and CD86, thereby delivering inhibitory signals that suppress T cell activation and proliferation (12). CTLA-4 inhibits IL-2 synthesis and cell cycle progression, and contributes to the termination of T-cell responses (13). Additionally, CTLA-4 can mediate the removal of immunostimulatory ligands from antigen-presenting cells through trogocytosis (14). Engagement of CTLA-4 on regulatory T cells enhances their immunosuppressive function. Thus, CTLA-4 inhibitors could enhance T cell activity by blocking CTLA-4, promoting T cell proliferation and cytokine secretion, activating self-reactive T cells, and consequently inducing T cell-mediated immune damage (15).

Programmed cell death protein 1 (PD-1) signaling is mediated by the tyrosine phosphatase SH2-containing protein tyrosine phos-phatase-1/2 (SHP-1/2), which dephosphorylates downstream molecules in the T cell receptor signaling pathway. Upon binding to its ligands—PD-L1 (also known as B7-H1 or CD274) and PD-L2 (also known as B7-DC or CD273), PD-1 inhibits kinases involved in T cell activation through recruitment of SHP-1 and SHP-2, thereby preventing self-tissue damage caused by excessive immune responses (14, 16). In addition to promoting the activity of these phosphatases, which dephosphorylate key signaling nodes essential for T cell co-stimulation, PD-1 has also been shown to suppress the RAS–extracellular signal-regulated kinase (ERK) pathway, a critical downstream cascade of TCR signaling (17). Tumor cells can evade immune surveillance by overexpressing PD-L1, which engages PD-1 on T cells and leads to suppression of T cell function, resulting in impaired recognition and elimination of tumor cells (18). Consequently, PD-1 inhibitors reverse T cell exhaustion and restore cytotoxic T cell function by blocking the interaction between PD-1 and its ligands, PD-L1 and PD-L2.

**Figure 1:**
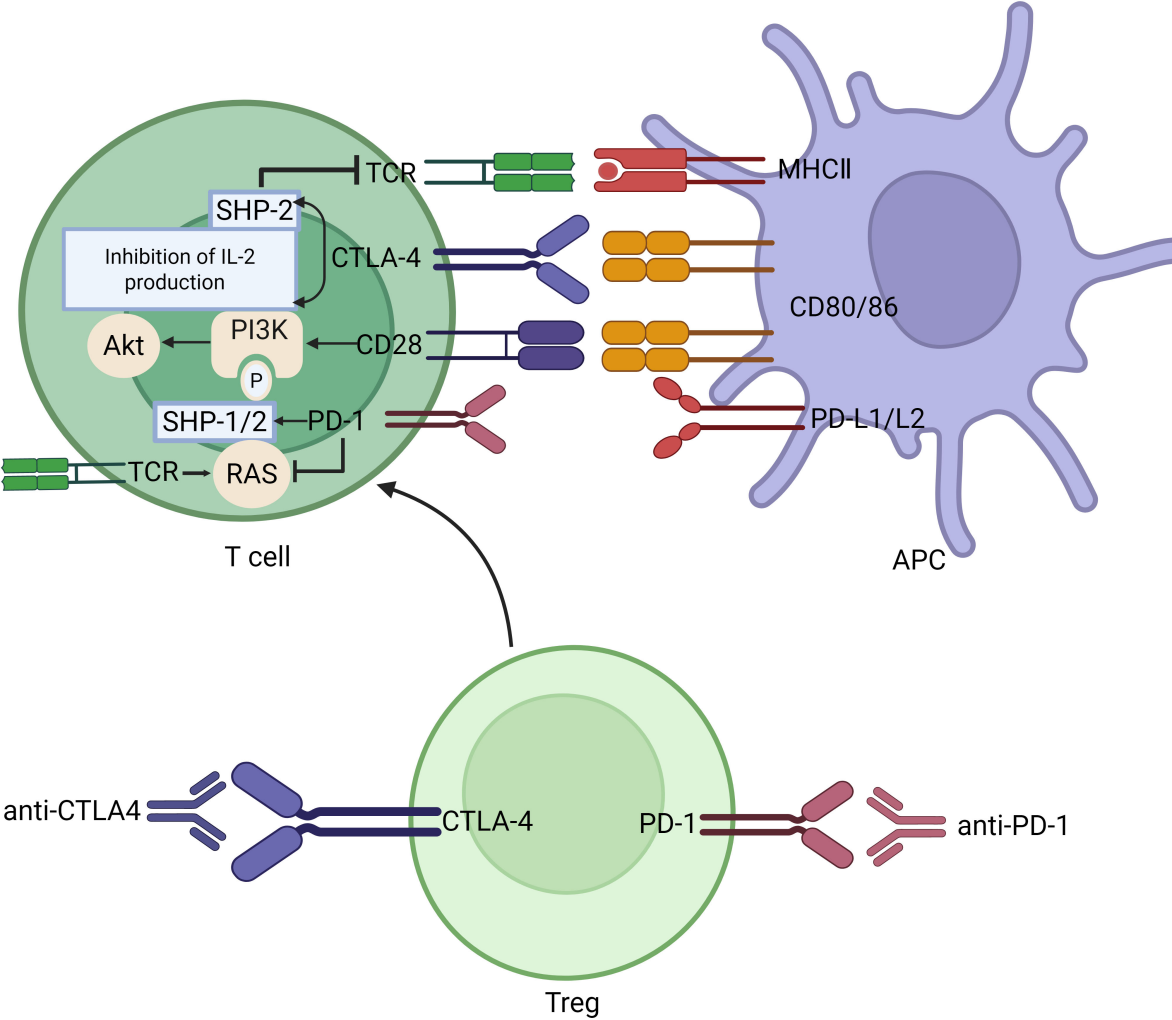
The antitumor mechanism of immune checkpoint inhibitors. When anti-PD-1/PD-L1 antibodies block the interaction between PD-L1 and T cell PD-1 receptors, SHP-1/2 kinase is no longer recruited to inhibit TCR signaling. In the meanwhile, RAS gene can be enhanced. CTLA-4 competes with CD28 for binding to CD80/CD86 on antigen-presenting cells. Anti-CTLA-4 antibodies promote the interaction between CD28 and CD80/CD86, enhancing PI3K-AKT-mTOR as well as restoring the cell cycle of T cells (IL-2 involved). Similar to anti-PD-1/PD-L1, SHP-2 kinase is no longer recruited to inhibit TCR signaling. Meanwhile, these inhibitors block regulatory T cells from binding to ligand, thus suppressing their ability to inhibit excessive T cell activation. PD-1, Programmed cell death protein-1; PD-L1, Programmed cell death ligand1; CTLA4, Cytotoxic T lymphocyte-associated antigen-4; TCR, T cell receptor; Treg, regulatory T cell; MHC, Major histocompatibility complex; APC, Antigen-presenting cell; SHP, SH2 domain-containing protein-tyrosine phosphatase. Created in BioRender. lv, w. (2026) https://BioRender.com/lcnbhgk.

Therefore, when ICIs are used, the immune system loses its ability to self-regulate, leading to a breakdown in immunologic tolerance toward self-tissues and resulting in immune-related adverse events (irAEs) (19). (The antitumor mechanism of immune checkpoint inhibitors is illustrated in **Figure 1**).

## 3 Epidemiology and clinical manifestation of ICIs-AKI

ICIs have significantly improved objective response rates and overall survival outcomes across multiple cancer types. However, ICIs are capable of inducing induce irAEs across multiple organ systems. A cohort study demonstrated that renal irAEs may co-occur with multiple extrarenal irAEs, particularly those affecting the endocrine and gastrointestinal systems (20). The overall incidence of irAEs associated with ICIs ranged from 45% to 83% (21) (any grade), with nephrotoxicity occurring in approximately 1% to 5% of cases, as well as ureteral and cystitis manifestations related to ICIs were rare (22, 23). Among patients with ICIs-AKI, more than one-third progressed to the most severe form of acute kidney injury, classified as AKI stage 3 (24, 25).

Over 80-90% of ICIs-AKI are acute tubulointerstitial nephritis (AIN) (25–27), characterized by CD4^+^ interstitial lymphocyte infiltration with eosinophils or phlogocyte. Data from the French Pharmacovigilance Database covering the period from 1985 to 2020 indicate that acute tubular necrosis (ATN) was reported in 28.6% (18/63) of patients, with ATN most frequently occurring concomitantly with AIN in 12 out of 18 cases (66.7%). However, preexisting renal susceptibility in elderly hypertensive individuals may be clinically significant, especially considering their frequent exposure to diuretics, renin-angiotensin system inhibitors, nonsteroidal anti-inflammatory drugs, and radiographic contrast agents—all of which are recognized risk factors for ATN, particularly in settings such as hypovolemia (25). Other renal pathologic types of ICIs-related kidney disease include various glomerulonephritis such as Pauci-immune Glomerulonephritis (including ANCA-positive or negative glomerulonephritis) (28), granulomatous arteritis (29), sarcoidosis-like reaction (30), Podocytopathy (primarily minimal change nephropathy), IgA nephropathy (31), membranous nephropathy, AA amyloidosis, C3 nephropathy, and anti-glomerular basement membrane disease (32). ICIs-related renal tubular acidosis and Fanconi syndrome has been documented in case reports. PD-1 checkpoint inhibitor-induced ureteritis and cystitis have also been reported in case studies, frequently resulting in misdiagnosis as urinary tract infections or obstructions (33). The renal pathological findings associated with ICIs-related renal disease are summarized in **Table 2**.

**Table 2:** The renal pathological findings associated with ICIs-related renal disease.

Research type	Reference	The number of renal biopsy cases	The incidence of biopsy-based diagnosis (%)			
			AIN	ATN	Glomerular disease	Other cases, such as the combination of two pathological types
Case Report	(26)	13	92.3		TMA (8.3%)	
Multicentre study/retrospective study	(27)	60	93.3		ANCA-positive or negative (1.7%) C3 nephropathy (1.7%) Anti-GBM (1.7%)	1.7%
Retrospective cohort study	(25)	63	82.5	28.6	FSGS (1.6%) MPGN (1.6%)	4.8%
Retrospective study	(22)	8	100			
Multicenter cohort study/retrospective study	(32)	151	82.7	unavailable	ANCA-positive or negative (0.7%) MPGN, IgA nephropathy: unavailable TMA (0.7%) Amyloidosis (0.7%) FSGS (1.3%) MPGN (0.7%) C3nephropathy (0.7%) MCD (0.7%)	
Database analysis (only Glomerular diseases)	(34)	53			ANCA-positive or negative (28.3%) IgA nephropathy (11.3%) Membranous nephropathy (7.5%) FSGS (3.8%) Amyloidosis (7.5%) C3 nephropathy (7.5%) Anti-GBM (3.8%) MCD (22.6%)	7.5%
Case Report	(31)	1			IgA nephropathy	
Case Report	(29)	1			Granulomatous arteritis	
Case Report	(30)	1			Sarcoid-like reaction	
Case Report	(28)	4			ANCA-positive or negative (25%) Renal Vasculitis (75%)	
Meta-analysis (glomerulopathy)	(141)	45			Podocytopathies (Minimal Change Disease and FSGS) (24%) AA amyloidosis (8.9%) Membranous Nephropathy (2.2%) Pauci-immune vasculitis (26.7%) C3 glomerulopathy (11.1%) IgA nephropathy (8.9%) Anti GBM disease (6.7%) Thrombotic microangiopathy (4.4%) Immune complex GN (4.4%) Lupus like Nephritis (2.2%)	

Correct to 1 demical place.

GN, glomerulopathy; AIN, Acute interstitial nephritis; ATN, acute tubular necrosis; TMA, thrombotic microangiopathy; anti-GBM, Anti-glomerular basement membrane; FSGS, focal segmental glomerular sclerosis; MPGN, Membranoproliferative Glomerulonephritis; MCD, minimal change disease.

ICIs-AIN predominantly occurs 3–4 months after the initiation of ICIs therapy (2, 36), manifesting as an increase in serum creatinine exceeding 50% of baseline renal function, and is frequently accompanied by sterile pyuria, oliguria, and edema, although the classic “triple sign” (fever, rash, eosinophilia) remains uncommon (35). The patients with ICI- associated glomerulonephritis mostly present with proteinuria, hematuria, edema, and hypertension (34). Refractory hypokalemia, metabolic acidosis, and Fanconi syndrome have been reported in patients with immune-mediated renal tubular acidosis (37, 38). However, electrolyte disturbances should prompt exclusion of immune-mediated endocrine gland injury (39). More recently, urinary irritation, sterile pyuria, gross hematuria, hydronephrosis, and ureteral dilation have been described in association with immune-mediated ureteritis and cystitis (22, 23).

A low baseline estimated glomerular filtration rate had increased ICIs-AKI risk (40, 41).The concurrent use of nonsteroidal anti-inflammatory drugs, renin-angiotensin-aldosterone system inhibitors, fluindione (42), high-dose diuretics, and the occurrence of extra-renal irAEs were significantly associated with ICIs-AKI (43). Notably, proton pump inhibitor was identified as an independent risk factor for ICI-AIN (44). Furthermore, the administration of ICI agents may inadvertently predispose individuals to an adverse immunological state characterized by a heightened propensity for hypersensitivity reactions, potentially exacerbating other drug-associated AKI (45). Additionally, analysis of 19,609,804 irAEs reports from the FDA Adverse Event Reporting System database revealed an 80% to 160% increased risk of developing irAEs among individuals who experienced concomitant infections during treatment with ICIs. Proposed mechanisms by which infectious agents may contribute to irAEs include cryptic antigen presentation, bystander activation, molecular mimicry, epitope spreading, and infection-induced T-cell autoreactivity (46).

Notably, the risk of ICIs-AKI was significantly higher with combination therapy—specifically, the use of anti-PD-1/PD-L1 agents in conjunction with anti-CTLA-4 agents—than with monotherapy (47).

Therefore, ICIs-AKI, predominantly immune-mediated AIN, is not an uncommon irAE, particularly in the context of concomitant use of nephrotoxic agents, underlying infections, or combination therapy with anti-PD-1/PD-L1 and anti-CTLA-4. ICIs-AKI can also be accompanied by immune-mediated ureteritis and cystitis. Severe hypokalemia in the absence of immune-related endocrine injury should prompt consideration of renal tubular acidosis. In cases of significant proteinuria or steroid resistance, early evaluation of renal pathology is essential to optimize renal prognosis.

## 4 Pathogenesis of ICIs-AKI

Single-cell RNA sequencing combined with mass cytometry conducted in 162 patients experiencing irAEs demonstrated that a preexisting activated, autoimmune-like inflammatory milieu plays a key role in the development of irAEs during ICIs treatment. This pathogenic process is driven by three core mechanisms: increased plasmablast activation and elevated anti-nuclear antibody titers, upregulated interferon-γ (INF-γ)/C-X-C motif chemokine ligand (CXCL)10/C-X-C chemokine receptor (CXCR)3 signaling pathway, and amplified tumor necrosis factor (TNF) signaling activity (48). The transcriptomic assessment of kidney tissue indicated that the genes most markedly overexpressed in ICI-AIN were predominantly linked to pathways involved in antigen processing and presentation, as well as those driving T cell-dependent immune activation (49, 50). Furthermore, renal pathology from patients with ICI-AIN reveal an accumulation of resident macrophages, fibroblasts, and CD8^+^ T cells, where the resident macrophages display heightened expression of pro-inflammatory and pro-fibrotic markers. In mouse models, administration of anti-PD-1 treatment induces renal injury characterized by immune cell infiltration, tubular impairment, and fibrosis. Importantly, eliminating tissue-resident macrophages reduces the levels of CXCL9 and matrix metalloproteinase 12, thereby alleviating kidney damage (51). Analysis of urinary and plasma proteomes identified Janus kinase-signal transducer and activator of transcription (JAK-STAT) and TNF signaling pathways as key contributors to the development of ICI-AIN (52).

**Figure 2:**
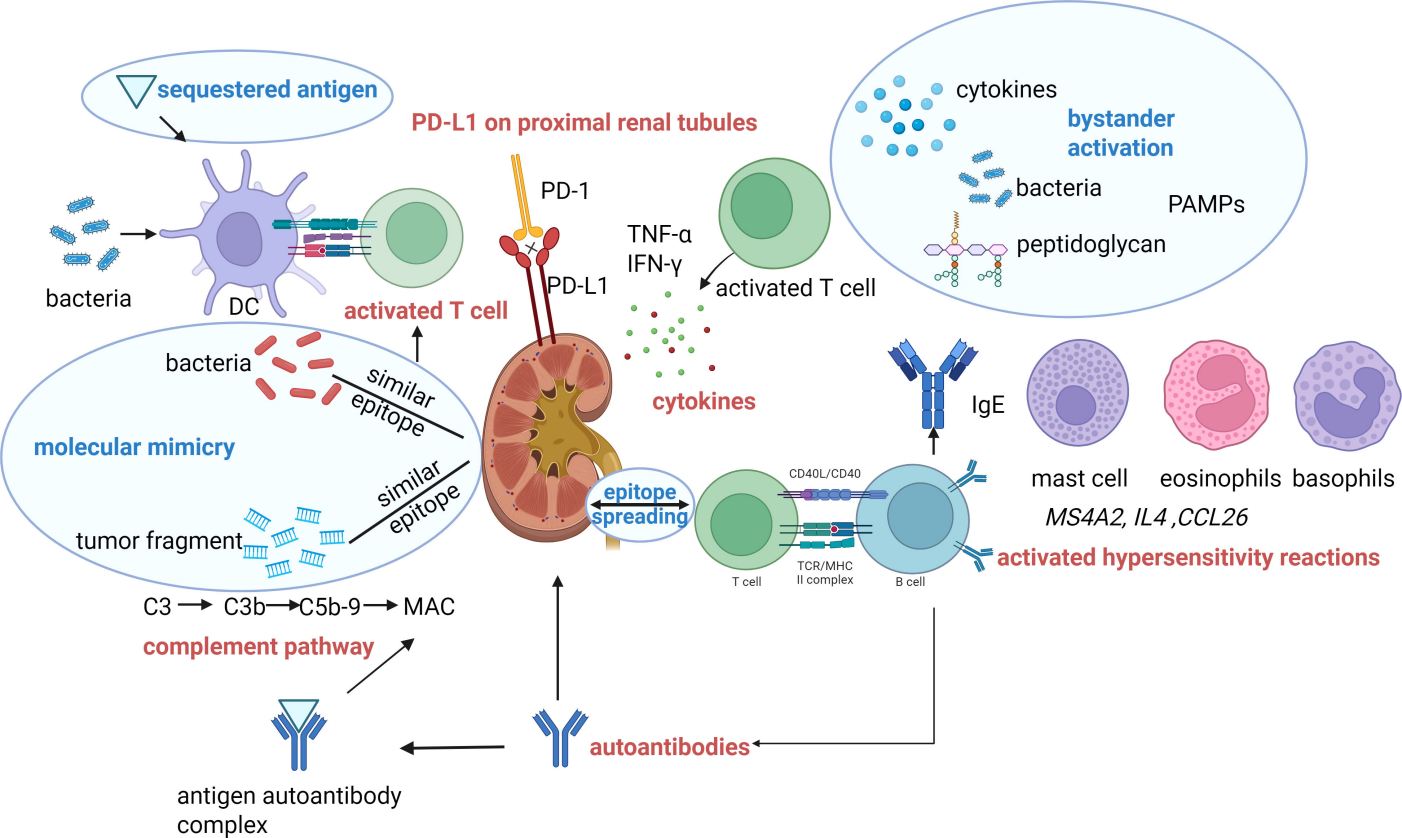
The proposed mechanisms underlying ICIs-induced AKI. Activated T-cells and inflammatory cytokines: T cells are activated as a result of disrupted immune tolerance—through mechanisms such as cryptic antigen presentation, bystander activation, molecular mimicry, and epitope spreading—leading to the subsequent release of inflammatory cytokines. Protective effect of PD-L1 on proximal renal tubules: Anti-PD-1/PD-L1 antibodies can block the interaction between PD-L1 expressed on renal tubular cells and PD-1 present on T cells, thereby disrupting immune tolerance and leading to immune-mediated renal tubular injury. Activated hypersensitivity reactions: *MS4A2* (membrane-spanning 4-domains subfamily A member 2), *IL4* (interleukin-4), and *CCL26* (C-C motif chemokine ligand 26)—genes known to be involved in allergic response pathways, including those linked to immunoglobulin E (IgE), mast cells, eosinophils, and basophils. Activated B cells produce Aabs: T cells release signals that activate B cells, which then produce autoantibodies that damage kidney tissue. Activated complement pathway: Autoantibodies and renal autoantigens form antigen-antibody complexes, which activate the complement pathway. DC, Dendritic cells; MAC, Membrane attack complex; TCR, T cell receptor; MHC, Major histocompatibility complex. PAMPs, Pathogen-Associated Molecular Patterns. Created in BioRender. lv, w. (2026) https://BioRender.com/sgsw389.

Several potential mechanisms underlying the pathophysiology of ICI-AKI are summarized below (**Figure 2**).

### 4.1 Active T-cells and inflammatory cytokines

The two immune checkpoints, CTLA-4 and PD-1/PD-L1, play complementary roles in maintaining peripheral immune tolerance (53). The blockade of either pathways removes the multilayered inhibition on self-reactive T cells, thereby enabling direct CD4^+^ or CD8^+^ T cell-mediated attacks on autologous tissues (54)—including renal tubular epithelium-and promoting cytokine—driven inflammatory amplification, ultimately resulting in immune-mediated AIN (55, 56).

Activated T cells can induce the release of inflammatory cytokines, such as IFN-γ and interleukin-21 (IL-21), which may play a role in the pathogenesis of ANCA-associated vasculitis (34).

Moreover, studies have shown that T lymphocyte infiltration is the predominant histopathological feature in the bladder tissues of most patients with ICIs-related ureteral cystitis (33). This inflammation of the urinary tract may occur concurrently with renal immune-related adverse events, suggesting a potential role of T cells in the pathogenesis of ICI-associated urinary system injury.

### 4.2 Activated hypersensitivity reactions

Transcriptomic analysis of renal tissue revealed that the genes most markedly overexpressed in ICI-AIN encompassed *MS4A2* (membrane-spanning 4-domains subfamily A member 2), *IL4* (interleukin-4), and *CCL26* (C-C motif chemokine ligand 26)—genes known to be involved in allergic response pathways, including those linked to immunoglobulin E (IgE), mast cells, eosinophils, and basophils (49). Therefore, ICIs-AIN may be induced or may facilitate acute renal tubulointerstitial injury caused by the concomitant use of nephrotoxic agents and underlying infections.

### 4.3 Autoantibody produced by activated B cells

T cells deliver helper signals that activate B cells, which subsequently produce autoantibody (Aabs) targeting autoantigens on tubular epithelial cells, mesangial cells, or podocytes (54). For example, patients treated with ipilimumab have been reported to develop nephrotic syndrome and lupus-like immune complex glomerular lesions, with the concurrent presence of anti-CTLA-4 antibodies, anti-double-stranded DNA antibodies, and antinuclear antibodies (57). Relevantly, ipilimumab treatment was associated to a lupus-like glomerulopathy, and to serum circulating levels of anti dsDNA and anti-nuclear antigen antibodies closely resembling the autoimmune lupus nephritis phenotype (58).

### 4.4 Protective effect of PD-L1 on proximal renal tubules

PD-L1 is constitutively expressed at low levels in the proximal renal tubules of normal kidney tissue. *In vitro* studies have shown that IFN-γ can upregulate PD-L1 expression in human proximal renal tubule epithelial cells (59, 60). The interaction between PD-L1 on renal tubular cells and PD-1 on T cells contributes to immune tolerance by protecting tubular cells from T cell-mediated immune damage (59, 61). However, binding of monoclonal antibodies to PD-L1 expressed on renal tubules may disrupt this protective mechanism, potentially resulting in tubular injury.

### 4.5 Activation of the complement pathway

ICIs may induce disorders of the complement system, such as the previously reported cases involving anti-C3b antibodies and C3 glomerulopathy (34). The Aabs mentioned may bind to autoantigens, subsequently forming antigen-antibody complexes that deposit in the glomeruli, thereby activating the complement cascade. T cell-mediated immune responses and complement activation represent the primary immunopathological mechanisms underlying drug-induced AIN (62). The role of complement activation in the pathogenesis of ICIs–induced glomerulonephropathy and AIN warrants further investigation.

Collectively, it was previously believed that activated T cells, resulting from disrupted immune tolerance-through mechanisms such as cryptic antigen presentation, bystander activation, molecular mimicry, and epitope spreading—and the subsequent release of inflammatory cytokines play a crucial role in the pathogenesis of ICIs-AIN. Emerging genetic and proteomic evidence has demonstrated that resident macrophages exhibiting heightened expression of pro-inflammatory and pro-fibrotic characteristics also contribute to disease development. The pathogenesis of glomerulonephritis in patients receiving ICIs needs further research; however, activation of T cells, B cells, and the complement cascade may collectively induce injury to intrinsic glomerular cells.

## 5 Noninvasive methods for the prediction or diagnosis for ICIs-AKI

Renal pathology obtained through renal biopsy serves as the gold standard for diagnosing renal irAEs. Given the clinical condition of patients with advanced malignancies, renal biopsy may not be a feasible or timely diagnostic approach. Therefore, the identification of non-invasive biomarkers is of critical importance, particularly in patients presenting with contraindications to renal biopsy, such as a solitary kidney or thrombocytopenia. Predictive biomarkers are listed in **Table 3**.

**Table 3:** Predictive biomarkers for ICIs-AKI.

Biomarkers		Disease evaluated	Cancer type	Treatment	Type of study	Experimental subject	Significance	Reference
Cytokines and their receptors	Urinary CXCL9	biopsy-confirmed ICI-AIN	non-specific, mainly lung cancer	anti-PD-1/L1 therapy/combination	retrospective study	homo sapiens	Strongly associated with ICI–AIN; Helpful to differentiate ICI-AIN from AKI-other.	(63)
	Urinary IL-9	biopsy-confirmed AIN	not applicable	not applicable	retrospective study	homo sapiens	Helpful to differentiate AIN with other pathological type	(64)
	Urinary TNF-α and IL-10	biopsy-confirmed ICI-AIN	non-specific, mainly Melanoma	anti-PD-1/L1 therapy anti-CTLA-4 therapy	prospective study	homo sapiens	Helpful to differentiate ICI-AIN from AKI-other.	(65)
	Urinary CXCL11, IL-6	biopsy-confirmed AIN	not applicable	not applicable	observational study	homo sapiens	Helpful to differentiate AIN with other pathological type	(66)
	Urinary MCP-1	AIN development in mice; Patients:AIN-diagnosed	Mice:not applicable Patients:Non-specific	cisplatin and anti-PDL1	Mice:Randomized Controlled Trial Patients:retrospective study	homo sapiens/C57BL6J mice	Helpful to differentiate ICI-AIN from ICI-ATN.	(67)
	Urinary IL-5+urinary Fas	biopsy-confirmed AIN	non-specific, mainly Melanoma	anti-PD-1/L1 therapy anti-CTLA-4 therapy	retrospective study	homo sapiens	AUC0.94 for diagnosing ICI-AIN, Helpful to differentiate ICI-AIN from other ICI-AKI.	(52)
	sIL-2R	biopsy-confirmed ICI-AIN or clinically adjudicated ICI-nephritis	non-specific, mainly lung cancer	anti-PD-1/L1 therapy anti-CTLA-4/PD-1 combination	retrospective cohort	homo sapiens	sIL-2R level in peripheral blood was significantly higher in patients with ICI-AKI	(68)
Immune cells	CD163^+^ M2 macrophage in kidney biopsy and urine	biopsy-proven ICI-AKI	non-specific, mainly Lung adenocarcinoma	Anti-PD-1/L1 therapy Anti-CTLA-4/PD-1 combination	cross-sectional and follow-up study	homo sapiens	Distinguish AIN from ATN in kidney biopsy CD163-M are detected in ICI-AIN and correlate both with severity at diagnosis and better prognosis at 3 months	(69–71)
	Serum CD45RA^+^CD8^+^ T cells, reduced memory-type CD27^+^CD19^+^ B cells	biopsy-confirmed ICI-AIN or clinically adjudicated ICI-nephritis	non-specific, mainly lung cancer	Anti-PD-1/L1 therapy Anti-CTLA-4/PD-1 combination	retrospective cohort	homo sapiens	cannot be generalized	(68)
	Urinary T cells	biopsy-confirmed ICI-AIN or Vasculitis	Non-specific	Anti-PD-1/L1 therapy Anti-CTLA-4/PD-1 combination	retrospective study	homo sapiens	Increase in patients with ICI-AKI	(72)
Others	Urinary and serum soluble PD-1	biopsy-confirmed ICI-AIN	not mentioned	not mentioned	retrospective study	homo sapiens	Helpful to differentiate ICI-AIN from other ICI-AKI.	(73)
	CRP & uRBP/Cr	met clinical criteria or biopsy proven ICI-AIN	Non-specific, mainly lung cancer	Anti-PD-1/L1 therapy Anti-CTLA-4 therapy Combination	retrospective study	homo sapiens	Helpful to differentiate ICI-AIN from AKI-other	(74)

This table specifically covers biomarkers for ICI-AKI, other potential markers for differentiation have been discussed in the text.

CXCL9, C-X-C motif chemokine ligand 9; IL, Interleukin; TNF-α, Tumor necrosis factor-α; MCP-1, monocyte chemotactic protein-1; sIL-2R, soluble interleukin-2 receptor.

### 5.1 Cytokines and their receptors

Several non-invasive biomarkers of ICIs-AKI have been reported in recent years, primarily including cytokines and their corresponding receptors. Urinary CXCL9, a chemokine associated with IFN-γ-related genes, is considered a promising novel biomarker for differentiating ICIs-AIN from other etiologies of AKI (63, 75). Recent studies demonstrated that elevated urinary levels of TNF-α (65), IL-9 (64) and IL-10 (65) may serve as potential biomarkers for differentiating ICIs–AIN from other etiologies of AKI. Urine proteomic analysis identified a biomarker signature—urinary IL-5 and urinary Fas—that achieved an area under the curve of 0.94 for the diagnosis of ICI-AIN (52).

The diagnostic specificity for ICI-AKI was 100% when the levels of soluble interleukin-2 receptor (sIL-2R) was more than 1.75 times the upper limit of normal (75). However, sIL-2R was also elevated in hematological malignancies and renal cell carcinoma (68). Therefore, these conditions should be clinically excluded when interpreting sIL-2R as a biomarker. IL-17 is primarily secreted by Th17 CD4^+^ cells, which are potent mediators of autoimmunity and are regulated by CTLA-4 (76). It has been reported not only to be associated with an increased incidence of irAEs, but also to be overexpressed during AKI (59), suggesting its potential as a promising biomarker for ICIs-AKI (77). Other cytokines, including urinary CXCL11, IL-6, and monocyte chemotactic protein-1 (66, 67), are currently under investigation for their potential to enable early identification of ICIs-AKI.

### 5.2 Immune cells

An elevation in urinary soluble CD163 levels was also observed during anti-PD-1 treatment in patients with melanoma (69). The predominance of urinary M1 macrophages is indicative of acute kidney injury secondary to AIN, whereas the dominance of urinary M2 macrophages may serve as a potential biomarker for disease progression, particularly in cases of crescentic glomerulonephritis. Urinary macrophage subtypes may therefore aid in distinguishing between renal interstitial and glomerular lesions (77). The detection rate of eosinophiluria is relatively low; however, its presence may enhance the pre-test probability of ICI-AIN (22, 78).

Compared with patients who did not develop ICI-AKI following ICI therapy, those with ICIs–AKI exhibited significantly lower absolute counts of CD8^+^ T cells and naïve CD45RA^+^CD8^+^ T cells in peripheral blood, along with reduced absolute counts of memory-type CD27^+^CD19^+^ B cells and a trend toward relative expansion of plasmablasts (68). Urinary T cells have been identified in patients with ICIs–AKI (ICI-AIN or Vasculitis), and these cells are typically clonally identical to renal T cell infiltrates, suggesting that urinary T cells may serve as a noninvasive biomarker for ICI-induced immune nephritis (72).

### 5.3 Other biomarkers

Serum C-reactive protein (CRP) and the urinary retinol-binding protein-to-creatinine ratio (uRBP/Cr) have been identified as potential biomarkers for differentiating ICIs-AKI from other causes of AKI (74). Although uRBP/Cr and CRP levels may also be elevated in other forms of tubular injury, the concurrent elevation of both CRP and uRBP/Cr in the absence of identifiable infections or inflammatory conditions may indicate the presence of ICI-AIN. Conversely, if both CRP and uRBP/Cr levels remain within normal ranges without requiring immunosuppressive therapy, the probability of ICIs-AKI is markedly diminished (78).

Urinary soluble PD-1 and serum soluble PD-1 levels are significantly elevated in patients with ICI-AIN compared to those with acute tubular necrosis. In cancer patients, a urinary soluble PD-1 level below 129.3 pg/mL demonstrated a sensitivity of 71.43% and a specificity of 94.44% in differentiating acute tubular necrosis from ICI-AIN. Therefore, both urinary and serum soluble PD-1 may serve as potential biomarkers for distinguishing acute interstitial nephritis from acute tubular necrosis in oncology patients (73).

Granzyme B (a T cell activation marker)-specific positron emission tomography (PET) imaging demonstrated elevated renal uptake associated with renal irAEs, which markedly decreased following administration of the immunosuppressive agent dexamethasone. Histological analysis confirmed the presence of granzyme B and immune cell infiltrates, showing a strong correlation with increased PET signal intensity. These findings suggest that granzyme B-targeted PET imaging could serve as a potential non-invasive approach for detecting renal involvement in irAEs. The presence of granzyme B was also confirmed in renal samples from patients that presented with clinical renal irAEs. Whether renal granzyme B expression can serve as a biomarker of renal irAE need further research (79).

Furthermore, renal imaging detection may assist in the diagnosis of ICI-AIN. Shruti et al. reported that patients with ICI-AIN, including 3 renal biopsy-confirmed cases among 9 patients, exhibited a marked increase in mean standardized uptake value on 2-deoxy-2-[18F]fluoro-D-glucose positron emission tomography—computed tomography (FDG-PET/CT) from baseline to the time of AIN diagnosis, compared with other patients (80). Bilateral renal enlargement, new or increasing perinephric stranding, and bilateral wedge-shaped cortical areas of hypoenhancement were identified as characteristic imaging features of ICI-AIN (81).

Collectively, urinary CXCL9 and TNF-α are considered promising novel biomarkers for differentiating ICIs-AIN from other forms of AKI. Granzyme B -specific PET imaging has demonstrated the presence of granzyme B and immune cell infiltrates associated with renal irAEs, representing a promising non-invasive imaging approach for assessing the severity of activated immune cell infiltration in the kidney. However, these biomarkers remain in the clinical research phase and require further validation through clinical application.

### 5.4 Susceptibility gene

The identification of predisposing genetic factors may help prevent the occurrence of renal irAEs in high-risk patients with malignancy. One study confirmed that differentially expressed genes and their enriched pathways identified in CD4^+^ T cells (*FOS, RPS26, JUN*) and CD8^+^ T cells (*RPS26, TRBV25-1, JUN*) play important roles in the development of renal irAEs events associated with anti-PD-1 therapy (50). The rs16957301 variant in the PCCA gene may serve as a risk marker for ICI-AKI in Caucasian populations (82). However, none of the forementioned studies on the susceptibility genes associated with ICI-AKI were based on renal pathological confirmation. *IFI27*, an IFN-α–induced transcript, was identified and validated as a novel biomarker for differentiating ICI–associated T cell–mediated rejection from ICI-AIN (49).

In addition to renal adverse events, the anti-CTLA4 induced irAEs were associated with a genetic variant associated with high SYK expression. This genetic profile could serve as a baseline biomarker for risk of severe irAEs (grade 3-5) (83). A retrospective study reveals that the existence of the *PDCD1 PD-1.6* polymorphism (*G allele*) was associated with the occurrence of severe and multiple irAEs in patients with mRCC (84). A genetic screening for irAEs (including nephrotoxicity) revealed that the C allele of *MAPK1* rs3810610 was a risk predictor for irAEs of all grades. The A allele of *ADAD1* rs17388568 increased the risk of high-grade irAEs (50). These susceptibility gene associated with extrarenal organs are anticipated to represent a promising research direction for renal irAEs.

Collectively, limited evidence currently exists regarding susceptibility genes associated with renal irAEs. These findings have not yet been incorporated into clinical practice, as further studies are required to validate the association and investigate potential risks across diverse populations. Further research is needed to identify these genetic factors and their role in renal irAEs, with the aim of preventing such complications in susceptible populations.

## 6 Treatment and prognosis of ICIs-AKI

Patients with ICIs-AKI have a worse renal prognosis compared to those with AKI attributable to other etiologies (85). The primary therapeutic regimen includes discontinuation of ICIs and suspected nephrotoxic drugs (35). Clinical studies on ICIs-AKI therapy are shown in **Table 4**.

**Table 4:** Clinical studies on the management of ICIs-AKI.

Drug	Patient capacity	Treatment regimen and outcome distribution	Research type	Reference
Corticosteroids	429 cases of ICI-AKI (125 biopsy-proven cases of ICI-AIN among a cohort of 151 biopsied patients [82.7%])	The median initial corticosteroid dose was 60 mg in prednisone equivalent units (IQR 50–80). Patients were treated with corticosteroids for a median of 41 days (IQR 26–75) before tapering to ≤10 mg of prednisone (or the equivalent). A total of 22 patients (5.1%) were treated with additional or alternative immunosuppressive agents. Initiation of corticosteroids within 3 days of ICI-AKI was associated with a higher odd of renal recovery compared with later initiation.	a multicenter retrospective cohort study	(32)
	114 cases (35 biopsy-proven cases of ICI-AIN)	Upon diagnosis of AIN, all patients received prednisone at a starting dose of 60 mg, and the duration of treatment ranged from 1 week to 12 weeks. Twenty-nine patients (83%) achieved complete or partial renal response at 3 months after initiation of treatment for AIN, and six patients (17%) did not. Eleven patients (31%) had renal relapse after treatment for AIN.	retrospective study	(86)
	14 cases (35% biopsy-proven or 65% clinically suspected ICI-AIN)	The starting equivalent dose of prednisone was higher in those who had a CR versus a PR (median 0.77 mg/kg versus 0.66 mg/kg).	retrospective study	(35)
	165 cases of ICI-AKI	56 (34%) received a shorter duration of treatment and 109 (66%) received a longer duration. Five of 56 patients (8.9%) in the shorter duration group and 12 of 109 (11%) in the longer duration group developed recurrent ICI-AKI or died.	retrospective study	(87)
	119 cases of ICI-AKI among a cohort of 138 patients	The median time from doubling of SCr to initiation of steroids was 4 (IQR, 1–12) days. Intravenous pulse steroids were used in 36 (30%) patients. The median initial oral steroid dose in prednisone equivalent units was 60 (IQR, 60–80) mg/d. Eleven (9%) patients received additional immunosuppression beyond steroids. Complete, partial, or no kidney recovery occurred in 40%, 45%, and 15% of patients, respectively.	multicenter, retrospective cohort study	(27)
	12 cases (4 cases of biopsy-proven or 8 cases of clinically suspected ICI-AIN)	Steroid dose (mg/kg) (median, range) 1.0 [0–2.0] Duration of steroid use (weeks)(median, range)4 [0–8]Rate of CR and PR of AIN were 67% and 8%, respectively.	single-center retrospective study	(88)
	27 cases of ICI-AKI	After the development of ICI-AKI, patients who received corticosteroids had a greater rate of renal recovery than did those who did not (62% vs. 38%).	single-center retrospective cohort study	(89)
MMF	11 cases (4 cases of AIN)	5/11 patients receiving (MMF) demonstrated renal recovery, of whom 2 non-responders had biopsy-proven AIN and 1 responder had biopsy-proven AIN	a multicenter cohort study	(32)
	4 biopsy-proven cases of ICIs-AIN	all had partial renal responses	a multicenter study	(27)
Infliximab	10 (8 cases of biopsy-proven or 2 cases of clinically suspected ICI-AIN)	Infliximab-containing regimens were used to treat 10 patients with ICI-AIN. Four patients relapsing after Glucocorticoid therapy achieved durable and complete renal recovery, four patients experienced partial renal recovery, and two patients showed no improvement in kidney function.	retrospective study	(90)
Rituximab	5 cases (ANCA-positive or negative)	Treatment containing rituximab resulted in partial to complete kidney recovery and no vasculitis relapse.	case series	(91)
Eculizumab	2 cases of thrombotic microangiopathy	Two cases of thrombotic microangiopathy in multiple myeloma patients on carfilzomib-combination treatment had good responses to two months of eculizumab treatment.	case series	(92)
Janus kinase inhibitor	1 biopsy-proven case of ICIs-AIN	Tofacitinib enabled successful steroid tapering.	case report	(93)

CR, Complete recovery; PR, partial recovery; OR, Overall Survival; ICIs, immune checkpoint inhibitors; AKI, acute kidney injury; AIN, acute tubulointerstitial nephritis; IQR, interquartile range; MMF, mycophenolate mofetil.

With regard to discontinuation of ICIs, it is crucial to avoid unnecessary discontinuation of ICIs, particularly in the absence of alternative treatment options, and to guide therapy based on findings from renal biopsy or non-invasive urinary biomarker as discussed above, in future clinical practice (21). For patients with stage 2 AKI or higher severity, ICI should be discontinued immediately (94).

Glucocorticoids are recommended as the first-line treatment for ICIs-AIN. Earlier initiation of glucocorticoid therapy (within three days of onset), is more than twice as likely to be associated with renal recovery compared to delayed initiation, and this association is significantly correlated with improved overall survival outcomes (32, 94). Prednisone initiation is recommended at a dosage range of 0.8 to 1 mg/kg in patients diagnosed with ICIs-AKI (suspected ICIs-AIN without confirmation by renal pathology) (94). For patients with AKI stage 3, oral corticosteroids are recommended as a sequential therapy following intravenous pulse corticosteroid therapy. A slow corticosteroid tapering regimen is recommended to reduce the risk of disease recurrence, given the prolonged half-life of ICI and their association with sustained T cell activity (35). The total duration of glucocorticoid treatment is variable but ranges from 6 to 8 weeks in most studies (94). A multicenter retrospective cohort analysis of immunotherapy-naïve patients with advanced melanoma receiving combination therapy with ipilimumab and nivolumab, along with a smaller group treated with nivolumab and ipilimumab, demonstrated that a peak steroid dose exceeding 80 mg other than cumulative steroid dose was significantly linked to worse progression-free survival and overall survival (95). However, prolonged corticosteroid therapy may lead to adverse events, including infections, steroid-induced diabetes mellitus, and skeletal complications. Early discontinuation of corticosteroids may enable eligible patients to undergo rechallenge with ICIs therapy sooner, thereby improving the overall management of malignancy.

Some studies have shown that short-course corticosteroid regimen (≤28 days) does not increase the risk of recurrence or mortality compared with long-course regimen (29–84 days), and renal function recovery is comparable between the two groups (87). However, this therapeutic approach requires validation through randomized controlled trials and may not be generalizable to non-ICIs-AIN pathological subtypes.

Although AIN, a common renal irAEs induced by ICIs, is generally responsive to glucocorticoid therapy, approximately 20% of long-term survivors develop CKD or experience a significant decline in renal function. Furthermore, data from a large cohort study indicate that 1.9% of patients are steroid-refractory or could not be successfully tapered off the dosage, otherwise adverse reactions would recur (96). This is attributed to incomplete recovery from AKI, subclinical interstitial nephritis, and recurrent renal insults (40).

There remains limited clinical evidence to guide the management of steroid-refractory ICI-AIN. Recent case series indicate that infliximab may be an effective therapeutic option for patients with relapsing or refractory ICIs-AIN (90). SM Herrmann et al. reports case series in which serial monitoring of noninvasive biomarkers was used to direct steroid-sparing therapy with infliximab for biopsy-proven ICIs-AIN.

They trend a cytokine panel prior to each infliximab infusion to monitor TNF-α levels, which are frequently elevated at baseline and may correlate with treatment response. Biomarkers (TNF-α/Cr, CXCL9/Cr, RBP/Cr) were utilized to guide the timing and duration of therapy prior to the occurrence of significant elevations in serum creatinine (97). Prednisone is recommended to be given concomitantly with infliximab at least for the first 2 weeks, until TNF-α blockade is effective in patients with steroid-dependent ICIs-AIN (78, 97).

Janus kinase (JAK) signaling contributes to the pathophysiology of irAEs by establishing and perpetuating a pro-inflammatory environment. Tofacitinib, an oral JAK inhibitor, has enabled successful steroid tapering and may represent a therapeutic option for refractory ICIs-AIN (93). However, we should use it cautiously because it has been associated with adverse cardiovascular outcomes and secondary cancers (94). Mycophenolate mofetil also holds potential in treating ICIs-AIN (27, 32).

Case series have demonstrated that tocilizumab has a favorable safety profile and efficacy in the treatment of irAEs in patients with melanoma and lung cancer (98). However, there have been no reported cases of tocilizumab use in the treatment of refractory ICIs-AIN. Given the involvement of inflammatory cytokines in ICIs-AKI, the efficacy and safety of biologic agents targeting specific cytokines—such as inhibitors of IL-6, IL-17, IL-23, and IL-12—in the management of renal irAEs, particularly steroid-resistant cases, require further investigation (98).

Rituximab is recommended in patients who develop ICIs-related renal vasculitis (91). Complement inhibitors may represent a viable therapeutic option for patients with thrombotic microangiopathy caused by complement activation (94).

The use of azathioprine, cyclophosphamide, or cyclosporine in the management of ICI-AIN is currently not recommended due to the limited availability of evidence-based clinical data (94). Limited evidence demonstrated that immunosuppressive therapy may potentially accelerate the progression of underlying malignancies, thereby posing a critical challenge in the management of such patients (79). Plasmapheresis which aims to clear up Aabs has been reported to successfully treat critically ill patients with renal irAEs in case report (26).

Currently, glucocorticoids are the first-line treatment for ICI-AIN, but there exists drug tolerance and side effects. Therefore, further clinical investigation is warranted to establish the optimal dosage and duration of immunosuppressive therapy that effectively balances therapeutic efficacy with the risk of malignancy progression in patients experiencing renal irAEs. Based on the pathogenesis of ICI-AKI, further clinical studies are needed to comprehensively evaluate the efficacy and safety profiles of immunosuppressive and biologic agents, including infliximab, tocilizumab, and abatacept, in the context of renal irAEs.

The prognosis of ICIs-AKI involves two critical dimensions: recovery of renal function and survival outcomes associated with the underlying malignancy. Consequently, there is significant clinical concern regarding whether reinitiating ICI therapy may lead to exacerbated renal injury or precipitate severe irAEs. Fortunately, several studies proved that ICI rechallenge is generally associated with milder toxicity compared to the initial course of ICI therapy. Patients who experience disease progression following discontinuation of ICI treatment, or those who stopped therapy due to irAEs, may derive clinical benefit from reintroducing ICIs. While reinitiating ICI therapy seems feasible and tolerable under careful surveillance, the decision should be carefully considered by weighing the potential benefits of rechallenge, patient-specific comorbidities, and the risk of recurrence of irAEs (99–101).

## 7 The use of ICIs in patients with CKD and concomitant malignancy

The primary metabolic pathway of ICIs involves intracellular uptake and lysosomal degradation, which occurs via phagocytosis or target antigen-mediated drug disposition (102). Since ICIs are not primarily cleared through the renal system, the theoretical risk of drug accumulation in patients with CKD is considered to be low (102). A retrospective cohort study found no significant difference in overall treatment-related or irAEs between patients with normal renal function and those with impaired renal function (103). ICIs have demonstrated comparable efficacy to other antineoplastic therapies. The median treatment duration in patients with CKD and renal cell carcinoma was similar to that observed in individuals with normal eGFR (104).

ICI has not been linked to a higher risk of AKI or renal failure in individuals with advanced CKD (10). A small-sample study suggested that the overall incidence of irAEs among patients undergoing maintenance hemodialysis or peritoneal dialysis (32%) may be lower than that reported in the general population (40—60%) during ICI therapy (9).

Although patients undergoing hemodialysis exhibit compromised immune function as a result of diminished cell-mediated and humoral immune responses, clinical evidence indicates that the pembrolizumab, a PD-1 inhibitor, retains the capacity to effectively activating anti-tumor immune responses (105). Notably, no irAEs were observed during the 34-week treatment period or after discontinuation of therapy (105). Moreover, given the high molecular weight of ICIs, the likelihood of these agents being removed by dialysis is remarkably low (9); this theoretically poses no risk to the drug’s half-life or therapeutic efficacy.

In a large cohort study, the skin and gastrointestinal tract were the most commonly affected organ systems in patients with CKD or ESRD, consistent with the pattern observed in the general population (10, 102). Notably, the relatively high incidence of hematological adverse events in patients with ESRD contrasts with the low frequency reported in the general population. However, given that anemia and other hematologic abnormalities in patients undergoing dialysis (hemodialysis or peritoneal dialysis) may have multifactorial etiologies, this observation does not necessarily indicate that ICIs pose increased hematological risks in patients with ESRD (102).

A series of case reports has demonstrated that ICIs can be administered to patients undergoing dialysis—4 (21%) receiving peritoneal dialysis and 15 (79%) hemodialysis—at standard doses, with 42% of patients achieving long-term survival (>12 months) or remission, particularly among those with melanoma (9).

Hemodialysis patients inherently face a high risk of infection due to impaired immune function. Moreover, corticosteroid therapy required for the management of irAEs further increases susceptibility to infection (106), including viral or bacterial pneumonia and Pneumocystis jirovecii pneumonia (107). Therefore, close monitoring of lymphocyte counts, markers of humoral immunity, and infection-related biomarkers is essential during ICI therapy to enable early detection, prevention, and management of infectious complications.

Collectively, ICIs are neither renally cleared nor dialyzable, and can therefore be administered to patients with CKD or even ESRD at standard doses with maintained efficacy and safety. However, irAEs should be closely monitored in CKD patients, particularly hematological adverse events in those with ESRD. Notably, corticosteroid therapy used to manage irAEs may further increase the risk of infection.

## 8 The use of ICIs in kidney transplant recipients

Kidney transplantation is the most effective treatment for advanced kidney disease. Kidney transplant recipients (KTRs) have been observed to exhibit a two-to four-fold higher cancer incidence compared to those without a history of transplantation (108, 109). A nationwide cohort study using data from the Scottish Renal Registry, the Scottish Cancer Registry, and hospitalization records from 1997 to 2021 reported that the incidence of cancer among KTRs under the age of 40 was seven times higher than that in the general population; this elevated risk decreased to three times the baseline level among KTRs over the age of 60 (110). During periods of functional kidney grafts, the occurrence of infection-associated and immune-related malignancies is elevated relative to non-functional phases. These cancers include Kaposi sarcoma, non-Hodgkin’s lymphoma, Hodgkin’s lymphoma, lip cancer, non-epithelial skin cancers, and kidney cancer (111). Additionally, colorectal cancer has emerged as a malignancy of particular concern among KTRs (112). Notably, cancer ranks as the second most common cause of mortality in KTRs. Among 38,130 patients undergoing their first kidney transplantation, 970 (2.5%) were identified with incident cancer, and 38% of these individuals died within four years following cancer detection (113). The absolute risk of death with a functioning graft was notably elevated among KTRs diagnosed with lung cancer (78%), post-transplant lymphoproliferative disorder (38%), melanoma (35%), or colorectal cancer (49%) (113).

The administration of ICIs in this population remains complex due to concerns about reduced therapeutic effectiveness under concurrent immunosuppression, as well as an increased likelihood of allograft rejection. Recent case series have reported an objective response rate ranging from 27% to 45% (114–116), with the incidence of acute rejection in KTRs receiving ICIs estimated between 30% and 48% (116–118). Notably, allograft loss occurs in 65% to 70% of those experiencing rejection (116–118). A meta-analysis of 128 studies encompassing 243 KTRs treated with ICIs reported overall survival rates were 41.6% at 250 days and 16.4% at 500 days; and progression-free survival rates were 31.0% at 250 days and 11.3% at 500 days (119). Therefore, early diagnosis of rejection in kidney transplantation is critical for preserving renal function and has a significant impact on the prognosis of KTRs with cancer receiving ICI therapy. Kidney allograft rejection typically occurs approximately 3 weeks after the initiation of ICIs, in contrast to ICIs-AIN, which tends to develop around 14 weeks following ICI initiation (115, 116, 120). Two biomarkers—donor-derived cell-free DNA (dd-cfDNA) (121) and urinary levels of CXCL-10 (122)—may serve as tools for the early detection of acute allograft rejection in KTRs undergoing ICIs therapy. An increased proportion of CD45RA-expressing effector memory T cells among CD8^+^ T cells has been associated with a higher risk of graft failure (123). In contrast, a distinct expansion of granzyme B-producing regulatory B cells has been observed in KTRs exhibiting operational tolerance, along with a robust B-cell signature linked to a low risk of graft failure (124). Kidney allograft rejection is definitively diagnosed through biopsy, which remains the gold standard in clinical practice. Among reported cases confirmed by biopsy to have kidney allograft rejection, T-cell mediated rejection is the most commonly observed form, while combined mixed T-cell and antibody-mediated rejection can also occur (115, 116). Elevated renal expression of the *IFI27* gene (interferon-alpha inducible protein 27) has been identified as a potential biomarker for distinguishing kidney allograft rejection from those with ICIs-AIN (49).

Current clinical practice suggested that a dynamic steroid regimen combined with conversion to mTOR inhibitors may be associated with a reduced risk of kidney allograft rejection while simultaneously enabling an antitumor response (116, 125–127). In addition, anti-IL-6 therapy is currently under investigation in randomized controlled trials for the treatment of T-cell-mediated rejection and antibody-mediated rejection, given that IL-6 is a key proinflammatory cytokine whose elevated levels have been associated with irAEs and poorer oncologic outcomes in patients receiving ICIs therapy (128, 129).

Collectively, given the high incidence and mortality of malignant tumors among KTRs, early tumor screening should be prioritized to enable timely diagnosis and intervention. For those who develop malignancies post-transplantation, a comprehensive assessment of risks and benefits is essential prior to initiating ICI therapy. During ICI treatment, regular monitoring of biomarkers associated with graft rejection is recommended to ensure effective antitumor activity while preserving allograft function. Future research should focus on identifying non-invasive biomarkers capable of differentiating acute rejection from ICI-AIN at an early stage. To achieve sustained tumor control without compromising transplanted kidney function, optimal individualized strategies combining optimal immunosuppressive regimen and immunotherapeutic therapy require further validation through clinical studies.

## 9 Targeted immunotherapy with the potential to reduce the risk of irAEs

Although ICIs can induce antitumor responses, their simultaneous activation of the immune system may trigger immune-mediated adverse reactions involving multiple organ systems, potentially resulting in life-threatening complications. Therefore, the targeted delivery of ICIs may not only inhibit malignancy progression but also hold the potential to reduce irAEs.

PD-1 transfected macrophage membrane-derived nanoparticles (PMMNPs) can target both T cells and macrophages, selectively accumulating in tumor tissues to reduce the required dose of ICIs and associated side effects while preserving therapeutic efficacy. Animal studies have demonstrated that intravenous administration of PMMNPs does not induce hepatic and renal toxicity *in vivo (*130). Researchers have developed calcium phosphate nanoparticles for the co-delivery of anti-PD-L1 and CD73 inhibitors. This delivery strategy circumvents irAEs associated with anti-PD-L1 therapy while significantly enhancing treatment efficacy and reducing the required dosage by twenty-fold (131).

Another study functionalized the surface of Glycyrrhiza uralensis Fisch root-derived nanovesicles with antitumor properties by conjugating a PD-L1 antibody and encapsulating the STING pathway agonist DMXAA, thereby constructing a multifunctional, plant-derived nanocapsule designated GP@DMX NV. This system is capable of specifically targeting melanoma cells with elevated PD-L1 expression, thereby enabling efficient delivery of immunostimulatory agents to tumor sites.

STING agonists are essential for the type I interferon response, which is a prerequisite for antigen-presenting cells to differentiate and kill tumor cells (132). Furthermore, STING agonists demonstrate synergistic antitumor effects when combined with PD-1 inhibitors (133, 134). Although activation of the mtDNA-cGAS-STING signaling pathway contributes to ferroptosis in AKI (135, 136), glycyrrhiza uralensis fisch roots-derived nanovesicles can serve as delivery vehicles to promote intratumoral accumulation of STING agonists, thereby enhancing dendritic cell maturation while mitigating renal damage. This approach not only improves therapeutic efficacy but also reduces the toxic side effects associated with conventional combination therapies, demonstrating enhanced biosafety and biocompatibility (137).

A gelase-responsive nanoparticle enables targeted delivery of anti-PD-1 and the TGF-β receptor I inhibitor galunisertib to tumor tissues, effectively suppressing malignancy progression without observed renal side-effects (138).

Professor Lin’s team designed a novel nanoscale coordination polymer particle, Cu/TI, loaded with Cu²^+^ and PD-L1 inhibitor derivatives. The particle enables precise delivery of anticancer metal ions and small-molecule ICIs while exhibiting favorable biocompatibility (139).

In addition to nanoparticles, targeted delivery can also be achieved using engineered yeast. When the ICI variant was expressed in Saccharomyces boulardii, the engineered probiotic yeast effectively targeted the PD-1-PD-L1 axis and significantly reduced intestinal tumor burden in a murine model of colorectal cancer resistant to conventional ICI therapy, while simultaneously inhibiting tissue dissemination and tumor infiltration (140).

Collectively, various engineered materials have demonstrated significant potential for tumor-targeted drug delivery and enhanced antitumor efficacy. Although preclinical studies in animal models have confirmed the safety and effectiveness of these agents, further research and rigorous validation are necessary before clinical translation can be achieved. Research on targeted immunotherapy, as presented in **Table 5**.

**Table 5:** Research on targeted immunotherapy.

Material	Way to achieve targeted delivery	Other advantages	Experimental subject	Reference
PMMNPs	Through the interaction between LFA-1 and ICAM-1 overexpressed in tumor-inflamed endothelial cells	The membrane of PMMNPs is specifically engineered to deliver SIRPα, which binds to CD47 on tumor cells, thereby enhancing the phagocytic function of macrophages.	mice	(130)
Calcium Phosphate Nanoparticles	Through improved permeability and retention effects, while enabling pH-responsive degradation to release anti-PD-L1 in the acidic tumor microenvironment.	Co-delivery of anti-PD-L1 and APCP (a specific CD73 inhibitor); reduce drug dosage	mice	(131)
GP@DMX NV	By chemically modifying anti-PD-L1 onto the membrane surface of nanovesicles, these vesicles gain tumor cell selectivity.	Activates the STING pathway while inhibiting tumor growth through direct action of GC NV; enhances dendritic cell activity.	mice	(137)
GPNPs	Targeted drug release in gelatinase-rich tumor sites	Simultaneously administering anti-PD-1and TGF-β receptor I inhibitor	mice	(138)
Cu/TI The core consists of Cu(II), with the shell being an IOX1 (5-carboxyl-8-hydroxyquinoline) and triphenylphosphine (TPP) complex.	The triphenylphosphine derivative of 5-carboxy-8-hydroxyquinoline (PD-L1 inhibitor), acts as an ion carrier to deliver Cu2^+^ into cancer cell mitochondria, thereby inducing copper-dependent apoptosis.	Induce DAMPs; Promote dendritic cell maturation	mice	(139)
Sb	Integrate the compact yet functional ICI variant into the Sb secretion circuit	Short intestinal transit time	mice	(140)

PMMNPs, PD-1 transfected macrophage membrane derived nanoparticles; ICAM-1, intercellular adhesion molecule-1; LFA-1, Leukocyte function-associated antigen-1; SIRPα, signal regulatory protein α; APCP, ADP[α,β-CH_2_], AMP-CP; GC NV, Glycyrrhiza uralensis Fisch roots-derived nanovesicles; GPNP, gelase-responsive nanoparticle; Sb, Saccharomyces cerevisiae var. Boulardii; DAMPs, Damage-associated molecular patterns.

## 10 Conclusion

Collectively, renal immune-related adverse events (irAEs) are common among cancer patients receiving immunotherapy. Early diagnosis and timely, effective interventions can improve renal outcomes and overall survival. Urinary and serum biomarkers, along with certain imaging modalities, represent promising non-invasive approaches for the detection of immune checkpoint inhibitor (ICI) -associated tubulointerstitial nephritis (AIN). Moreover, the identification of genetic polymorphisms may help detect individuals carrying susceptible genotypes for renal irAEs, although this requires further investigation. Corticosteroids are the first-line treatment for ICI-AIN. Further clinical studies are warranted to determine the optimal dosage and duration of immunosuppressive therapy that balances efficacy with the risk of tumor progression in patients with renal irAEs. Infliximab is safe and effective in steroid-resistant cases. Additional research is needed to comprehensively evaluate the efficacy and safety of immunosuppressive and biologic agents—including tocilizumab, and Janus kinase inhibitors—in managing renal irAEs. Although ICI rechallenge appears safe under close monitoring, the decision should be individualized, weighing the potential benefits against patient comorbidities and the risk of irAE recurrence. Although ICIs are not renally excreted, severe kidney injury may compromise tolerance to subsequent chemotherapy or immunotherapy regimens. For renal transplant recipients, close monitoring is essential to prevent allograft rejection induced by ICIs. Biomarkers, along with a dynamic steroid regimen and conversion to mTOR inhibitors, represent critical strategies for mitigating the risk of kidney allograft rejection while simultaneously enabling an antitumor response. Furthermore, targeted immunotherapies for malignancies hold promise not only in controlling tumor progression but also in minimizing the incidence and severity of irAEs, thereby improving renal outcomes and survival prognosis in cancer patients.

## References

[1] De Giglio A Grandinetti V Aprile M Borelli G Campus A Croci Chiocchini AL . Patterns of renal toxicity from the combination of pemetrexed and pembrolizumab for advanced nonsquamous non-small-cell lung cancer (NSCLC): A single-center experience. Lung Cancer. (2022) 174:91–6. doi: 10.1016/j.lungcan.2022.10.007, PMID: 36356493

[2] Perazella MA Shirali AC . Immune checkpoint inhibitor nephrotoxicity: what do we know and what should we do? Kidney Int. (2020) 97:62–74. doi: 10.1016/j.kint.2019.07.022, PMID: 31685311

[3] Kitchlu A Reid J Jeyakumar N Dixon SN Munoz AM Silver SA . Cancer risk and mortality in patients with kidney disease: A population-based cohort study. Am J Kidney Dis. (2022) 80:436–448.e1. doi: 10.1053/j.ajkd.2022.02.020, PMID: 35405208

[4] Lees JS Elyan BMP Herrmann SM Lang NN Jones RJ Mark PB . The 'other' big complication: how chronic kidney disease impacts on cancer risks and outcomes. Nephrol Dial Transplant. (2023) 38:1071–9. doi: 10.1093/ndt/gfac011, PMID: 35090037 PMC10157781

[5] Tendulkar KK Cope B Dong J Plumb TJ Campbell WS Ganti AK . Risk of Malignancy in patients with chronic kidney disease. PloS One. (2022) 17:e0272910. doi: 10.1371/journal.pone.0272910, PMID: 35976968 PMC9385037

[6] Park S Lee S Kim Y Lee Y Kang MW Han K . Risk of cancer in pre-dialysis chronic kidney disease: A nationwide population-based study with a matched control group. Kidney Res Clin Pract. (2019) 38:60–70. doi: 10.23876/j.krcp.18.0131, PMID: 30866180 PMC6481964

[7] Butler AM Olshan AF Kshirsagar AV Edwards JK Nielsen ME Wheeler SB . Cancer incidence among US Medicare ESRD patients receiving hemodialysis, 1996–2009. Am J Kidney Dis. (2015) 65:763–72. doi: 10.1053/j.ajkd.2014.12.013, PMID: 25662835 PMC4924349

[8] Kitchlu A Silva V Anand S Kala J Abudayyeh A Inker LA . Assessment of GFR in patients with cancer: A statement from the american society of onco-nephrology. Clin J Am Soc Nephrol. (2024) 19:1061–72. doi: 10.2215/cjn.0000000000000508, PMID: 38848131 PMC11321742

[9] Strohbehn IA Lee M Seethapathy H Chute D Rahma O Guidon A . Safety and Efficacy of immune checkpoint inhibitors in patients on dialysis: A retrospective case series. Am J Kidney Dis. (2020) 76:299–302. doi: 10.1053/j.ajkd.2020.02.451, PMID: 32417401 PMC7997681

[10] Tiu BC Strohbehn IA Zhao S Ouyang T Hanna P Wang Q . Safety of immune checkpoint inhibitors in patients with advanced chronic kidney disease: A retrospective. Cohort Study Oncologist. (2023) 28:e379–90. doi: 10.1093/oncolo/oyad001, PMID: 36821637 PMC10243781

[11] Khan M Arooj S Wang H . NK cell-based immune checkpoint inhibition. Front Immunol. (2020) 11:167. doi: 10.3389/fimmu.2020.00167, PMID: 32117298 PMC7031489

[12] Hossen MM Ma Y Yin Z Xia Y Du J Huang JY . Current understanding of CTLA-4: from mechanism to autoimmune diseases. Front Immunol. (2023), 14:1198365. doi: 10.3389/fimmu.2023.1198365, PMID: 37497212 PMC10367421

[13] Sharpe AH Freeman GJ . The B7-CD28 superfamily. Nat Rev Immunol. (2002) 2:116–26. doi: 10.1038/nri727, PMID: 11910893

[14] Munir AZ Gutierrez A Qin J Lichtman AH Moslehi JJ . Immune-checkpoint inhibitor-mediated myocarditis: CTLA4, PD1 and LAG3 in the heart. Nat Rev Cancer. (2024) 2024:540–53:24(8). doi: 10.1038/s41568-024-00715-5, PMID: 38982146

[15] Chen L Flies DB . Molecular mechanisms of T cell co-stimulation and co-inhibition. Nat Rev Immunol. (2013) 13:227–42. doi: 10.1038/nri3405, PMID: 23470321 PMC3786574

[16] Ye Q Liu J Xie K . B7 family proteins in cancer progression: immunological and non-immunological functions. J Cancer Treat diagnosis. (2019). doi: 10.29245/2578-2967/2019/4.1171

[17] Ham H Jing H Lamborn IT Kober MM Koval A Berchiche YA . Germline mutations in a G protein identify signaling cross-talk in T cells. Science. (2024) 385:eadd8947. doi: 10.1126/science.add8947, PMID: 39298586 PMC11811912

[18] Liang Y Zheng Y Zeng Y Hu C Si Y Fan X . Immune checkpoint inhibitors in melanoma: mechanisms, immune cell interactions, and the tumour microenvironment. Front Immunol. (2025) 16:1691608. doi: 10.3389/fimmu.2025.1691608, PMID: 41346595 PMC12672547

[19] Kwiatkowska E Domański L Dziedziejko V Kajdy A Stefańska K Kwiatkowski S . The mechanism of drug nephrotoxicity and the methods for preventing kidney damage. Int J Mol Sci. (2021) 22:6109. doi: 10.3390/ijms22116109, PMID: 34204029 PMC8201165

[20] Wan G Chen W Khattab S Roster K Nguyen N Yan B . Multi-organ immune-related adverse events from immune checkpoint inhibitors and their downstream implications: a retrospective multicohort study. Lancet Oncol. (2024) 25:1053–69. doi: 10.1016/s1470-2045(24)00278-x, PMID: 39025103 PMC11316445

[21] Longhitano E Muscolino P Lo Re C Ferrara SA Cernaro V Gembillo G . Immune checkpoint inhibitors and the kidney: A focus on diagnosis and management for personalised medicine. Cancers (Basel). (2023) 15:1891. doi: 10.3390/cancers15061891, PMID: 36980777 PMC10046877

[22] Oleas D Bolufer M Agraz I Felip E Muñoz E Gabaldón A . Acute interstitial nephritis associated with immune checkpoint inhibitors: a single-centre experience. Clin Kidney J. (2021) 14:1364–70. doi: 10.1093/ckj/sfaa008, PMID: 34221369 PMC8247740

[23] Kommer A Stortz M Kraus D Weinmann-Menke J . Immune checkpoint inhibitor-associated acute kidney injury: A single-center experience of biopsy-proven cases. J Clin Med. (2025) 14:3231. doi: 10.3390/jcm14093231, PMID: 40364262 PMC12072658

[24] Esposito P Bottini A Lecini E Cappadona F Piaggio M Macciò L . Biopsy-proven acute tubulointerstitial nephritis in patients treated with immune checkpoint inhibitors: a pooled analysis of case reports. Front Oncol. (2023) 13:1221135. doi: 10.3389/fonc.2023.1221135, PMID: 37936605 PMC10627243

[25] Gérard AO Andreani M Fresse A Parassol N Muzzone M Pinel S . Immune checkpoint inhibitors-induced nephropathy: a French national survey. Cancer Immunol Immunother. (2021) 70:3357–64. doi: 10.1007/s00262-021-02983-8, PMID: 34155532 PMC10991556

[26] Cortazar FB Marrone KA Troxell ML Ralto KM Hoenig MP Brahmer JR . Clinicopathological features of acute kidney injury associated with immune checkpoint inhibitors. Kidney Int. (2016) 90:638–47. doi: 10.1016/j.kint.2016.04.008, PMID: 27282937 PMC4983464

[27] Cortazar FB Kibbelaar ZA Glezerman IG Abudayyeh A Mamlouk O Motwani SS . Clinical features and outcomes of immune checkpoint inhibitor-associated AKI: A multicenter study. J Am Soc Nephrol. (2020) 31:435–46. doi: 10.1681/asn.2019070676, PMID: 31896554 PMC7003302

[28] Gallan AJ Alexander E Reid P Kutuby F Chang A Henriksen KJ . Renal vasculitis and pauci-immune glomerulonephritis associated with immune checkpoint inhibitors. Am J Kidney Dis. (2019) 74:853–6. doi: 10.1053/j.ajkd.2019.04.016, PMID: 31204194

[29] Lemoine M Dilly B Curie A Hébert V Laurent C Hanoy M . Ipilimumab-induced renal granulomatous arteritis: a case report. BMC Nephrol. (2019) 20:366. doi: 10.1186/s12882-019-1552-2, PMID: 31604452 PMC6788031

[30] Park SD Kim MS Han MH Kim YJ Jung HY Choi JY . Renal sarcoidosis-like reaction induced by PD-1 inhibitor treatment in non-small cell lung cancer: A case report and literature review. Med (Kaunas). (2023) 59:991. doi: 10.3390/medicina59050991, PMID: 37241223 PMC10224123

[31] Wang R Das T Takou A . IgA nephropathy after pembrolizumab therapy for mesothelioma. BMJ Case Rep. (2020) 13:e237008. doi: 10.1136/bcr-2020-237008, PMID: 33257374 PMC7705558

[32] Gupta S Short SAP Sise ME Prosek JM Madhavan SM Soler MJ . Acute kidney injury in patients treated with immune checkpoint inhibitors. J Immunother Cancer. (2021) 9:e003467. doi: 10.1136/jitc-2021-003467, PMID: 34625513 PMC8496384

[33] Li S Zheng K Xu Y Wang M . Immune checkpoint inhibitors related cystoureteritis:A case report and literature review. Zhongguo Fei Ai Za Zhi. (2023) 26:709–16. doi: 10.3779/j.issn.1009-3419.2023.106.17, PMID: 37985157 PMC10600747

[34] He X Liu F Jin Y Fu H Mao J . Glomerular diseases after immune checkpoint inhibitors use: What do We know so far? Ren Fail. (2022) 44:2046–55. doi: 10.1080/0886022x.2022.2147439, PMID: 36420664 PMC9704066

[35] Manohar S Ghamrawi R Chengappa M Goksu BNB KottsChade L Finnes H . Acute interstitial nephritis and checkpoint inhibitor therapy: single center experience of management and drug rechallenge. Kidney 360. (2020) 1:16–24. doi: 10.34067/kid.0000152019, PMID: 35372854 PMC8808482

[36] Knox A Cloney T Janssen H Solomon BJ Alexander M Ruderman I . Immune-related acute kidney injury in Australian non-small cell lung cancer patients: Real-world results. Lung Cancer. (2023) 184:107325. doi: 10.1016/j.lungcan.2023.107325, PMID: 37573702

[37] Gu Y Yi L Zou X Guo L Wu G Zhao J . Refractory hypokalemia and metabolic acidosis induced by undifferentiated connective tissue disease secondary to immune checkpoint inhibitors: a case report and literature review. Front Oncol. (2024) 14:1442605. doi: 10.3389/fonc.2024.1442605, PMID: 39659778 PMC11628383

[38] Farid S Latif H Nilubol C Kim C . Immune checkpoint inhibitor-induced fanconi syndrome. Cureus. (2020) 12:e7686. doi: 10.7759/cureus.7686, PMID: 32431966 PMC7233517

[39] Terashita M Yazawa M Murakami N Nishiyama A . Water and electrolyte abnormalities in novel pharmacological agents for kidney disease and cancer. Clin Exp Nephrol. (2025) 29:521–33. doi: 10.1007/s10157-025-02635-6, PMID: 39937358 PMC12049309

[40] Seethapathy H Herrmann SM Sise ME . Immune checkpoint inhibitors and kidney toxicity: advances in diagnosis and management. Kidney Med. (2021) 3:1074–81. doi: 10.1016/j.xkme.2021.08.008, PMID: 34939017 PMC8664750

[41] Guven DC Ozbek DA Sahin TK Kavgaci G Aksun MS Erul E . The incidence and risk factors for acute kidney injury in patients treated with immune checkpoint inhibitors. Anticancer Drugs. (2023) 34:783–90. doi: 10.1097/cad.0000000000001463, PMID: 36729111

[42] Liu C Wei W Yang L Li J Yi C Pu Y . Incidence and risk factors of acute kidney injury in cancer patients treated with immune checkpoint inhibitors: a systematic review and meta-analysis. Front Immunol. (2023) 14:1173952. doi: 10.3389/fimmu.2023.1173952, PMID: 37313406 PMC10258324

[43] Liu K Qin Z Ge Y Bian A Xu X Wu B . Acute kidney injury in advanced lung cancer patients treated with PD-1 inhibitors: a single center observational study. J Cancer Res Clin Oncol. (2023) 149:5061–70. doi: 10.1007/s00432-022-04437-9, PMID: 36326913 PMC11797402

[44] Mohan A Krisanapan P Tangpanithandee S Thongprayoon C Kanduri SR Cheungpasitporn W . Association of proton pump inhibitor use and immune checkpoint inhibitor-mediated acute kidney injury: A meta-analysis and a review of related outcomes. Am J Nephrol. (2024) 55:439–49. doi: 10.1159/000538274, PMID: 38471492 PMC11870660

[45] Hammond S Olsson-Brown A Gardner J Thomson P Ali SE Jolly C . T cell mediated hypersensitivity to previously tolerated iodinated contrast media precipitated by introduction of atezolizumab. J Immunother Cancer. (2021) 9:e002521. doi: 10.1136/jitc-2021-002521, PMID: 34049931 PMC8166637

[46] Grabska S Grabski H Makunts T Abagyan R . Co-occurring infections in cancer patients treated with checkpoint inhibitors significantly increase the risk of immune-related adverse events. Cancers (Basel). (2024) 16:2820. doi: 10.3390/cancers16162820, PMID: 39199593 PMC11352782

[47] Chen P Zhu J Xu Y Huang Q Su J Gao Z . Risk factors of immune checkpoint inhibitor-associated acute kidney injury: evidence from clinical studies and FDA pharmacovigilance database. BMC Nephrol. (2023) 24:107. doi: 10.1186/s12882-023-03171-9, PMID: 37087434 PMC10122540

[48] Khan S Malladi VS von Itzstein MS Mu-Mosley H Fattah FJ Liu Y . Innate and adaptive immune features associated with immune-related adverse events. J Immunother Cancer. (2025) 13:e012414. doi: 10.1136/jitc-2025-012414, PMID: 40930749 PMC12506470

[49] Adam BA Murakami N Reid G Du K Jasim R Boils CL . Gene expression profiling in kidney transplants with immune checkpoint inhibitor-associated adverse events. Clin J Am Soc Nephrol. (2021) 16:1376–86. doi: 10.2215/cjn.00920121, PMID: 34244334 PMC8729568

[50] Liu S Lu P Yang B Yang Y Zhou H Yang M . Single-cell RNA sequencing analysis of peripheral blood mononuclear cells in PD-1-induced renal toxicity in patients with lung cancer. BMC Nephrol. (2024) 25:307. doi: 10.1186/s12882-024-03754-0, PMID: 39277735 PMC11401319

[51] Ma Y Chen Y Yao Q Wang Y Ouyang N Han F . Resident macrophage-orchestrated immune and fibroblast interactions in immune checkpoint inhibitor-associated nephrotoxicity. Adv Sci (Weinh). (2025) 12(42):e05445. doi: 10.1002/advs.202505445, PMID: 40810645 PMC12622469

[52] Long JP Singh S Dong Y Yee C Lin JS . Urine proteomics defines an immune checkpoint-associated nephritis signature. J Immunother Cancer. (2025) 13:e010680. doi: 10.1136/jitc-2024-010680, PMID: 39863302 PMC11784134

[53] Francisco LM Sage PT Sharpe AH . The PD-1 pathway in tolerance and autoimmunity. Immunol Rev. (2010) 236:219–42. doi: 10.1111/j.1600-065X.2010.00923.x, PMID: 20636820 PMC2919275

[54] Moturi K Sharma H Hashemi-Sadraei N . Nephrotoxicity in the age of immune checkpoint inhibitors: mechanisms, diagnosis, and management. Int J Mol Sci. (2023) 25:414. doi: 10.3390/ijms25010414, PMID: 38203586 PMC10778678

[55] Zehn D Bevan MJ . T. cells with low avidity for a tissue-restricted antigen routinely evade central and peripheral tolerance and cause autoimmunity. Immunity. (2006) 25:261–70. doi: 10.1016/j.immuni.2006.06.009, PMID: 16879996 PMC2774714

[56] Richards DM Kyewski B Feuerer M . Re-examining the nature and function of self-reactive T cells. Trends Immunol. (2016) 37:114–25. doi: 10.1016/j.it.2015.12.005, PMID: 26795134 PMC7611850

[57] Izzedine H Gueutin V Gharbi C Mateus C Robert C Routier E . Kidney injuries related to ipilimumab. Invest New Drugs. (2014) 32:769–73. doi: 10.1007/s10637-014-0092-7, PMID: 24687600

[58] Benfaremo D Manfredi L Luchetti MM Gabrielli A . Musculoskeletal and rheumatic diseases induced by immune checkpoint inhibitors: A review of the literature. Curr Drug Saf. (2018) 13:150–64. doi: 10.2174/1574886313666180508122332, PMID: 29745339 PMC6198478

[59] Franzin R Netti GS Spadaccino F Porta C Gesualdo L Stallone G . The use of immune checkpoint inhibitors in oncology and the occurrence of AKI: where do we stand? Front Immunol. (2020) 11:574271. doi: 10.3389/fimmu.2020.574271, PMID: 33162990 PMC7580288

[60] Ding H Wu X Gao W . PD-L1 is expressed by human renal tubular epithelial cells and suppresses T cell cytokine synthesis. Clin Immunol. (2005) 115:184–91. doi: 10.1016/j.clim.2005.01.005, PMID: 15885642

[61] Catalano M Roviello G Galli IC Santi R Nesi G . Immune checkpoint inhibitor induced nephrotoxicity: An ongoing challenge. Front Med (Lausanne). (2022) 9:1014257. doi: 10.3389/fmed.2022.1014257, PMID: 36606052 PMC9807763

[62] Karimzadeh I Barreto EF Kellum JA Awdishu L Murray PT Ostermann M . Moving toward a contemporary classification of drug-induced kidney disease. Crit Care. (2023) 27:435. doi: 10.1186/s13054-023-04720-2, PMID: 37946280 PMC10633929

[63] Gupta S Mistry K Alikhan FM Mejia SM Sadarangani S Cao A . Urinary C-X-C-motif ligand 9 (CXCL9) in immune checkpoint inhibitor-associated acute interstitial nephritis. Kidney Int. (2025) 108(3):491–6. doi: 10.1016/j.kint.2025.05.029, PMID: 40578686 PMC12341002

[64] Moledina DG Wilson FP Pober JS Perazella MA Singh N Luciano RL . TNF-α and IL-9 for clinical diagnosis of acute interstitial nephritis. JCI Insight. (2019) 4:e127456. doi: 10.1172/jci.insight.127456, PMID: 31092735 PMC6542610

[65] Farooqui N Zaidi M Vaughan L McKee TD Ahsan E Pavelko KD . Cytokines and immune cell phenotype in acute kidney injury associated with immune checkpoint inhibitors. Kidney Int Rep. (2023) 8:628–41. doi: 10.1016/j.ekir.2022.11.020, PMID: 36938084 PMC10014345

[66] Martinez Valenzuela L Draibe J Bestard O Fulladosa X Gómez-Preciado F Antón P . Urinary cytokines reflect renal inflammation in acute tubulointerstitial nephritis: A multiplex bead-based assay assessment. J Clin Med. (2021) 10:2986. doi: 10.3390/jcm10132986, PMID: 34279469 PMC8268986

[67] Martinez Valenzuela L Gómez-Preciado F Guiteras J Antón Pampols P Gomà M Fulladosa X . Immune checkpoint inhibitors induce acute interstitial nephritis in mice with increased urinary MCP1 and PD-1 glomerular expression. J Transl Med. (2024) 22:421. doi: 10.1186/s12967-024-05177-9, PMID: 38702780 PMC11069287

[68] Sise ME Wang Q Seethapathy H Moreno D Harden D Smith RN . Soluble and cell-based markers of immune checkpoint inhibitor-associated nephritis. J Immunother Cancer. (2023) 11:e006222. doi: 10.1136/jitc-2022-006222, PMID: 36657813 PMC9853261

[69] Perier T Renaudineau Y Pellegrini J Colombat M Ramirez AA Guy P . CD163 detection in immune check-point inhibitors-related acute interstitial nephritis. Clinical Kidney Journal. (2025) 18(3):sfaf009. doi: 10.1093/ckj/sfaf009, PMID: 40052170 PMC11883220

[70] Sun PP Zhou XJ Su JQ Wang C Yu XJ Su T . Urine macrophages reflect kidney macrophage content during acute tubular interstitial and glomerular injury. Clin Immunol. (2019) 205:65–74. doi: 10.1016/j.clim.2019.06.005, PMID: 31212026

[71] Kim MG Lim K Lee YJ Yang J Oh SW Cho WY . M2 macrophages predict worse long-term outcomes in human acute tubular necrosis. Sci Rep. (2020) 10:2122. doi: 10.1038/s41598-020-58725-w, PMID: 32034190 PMC7005727

[72] Singh S Clemente LC Parra ER Tchakarov A Yang C Li Y . Urinary T cells are detected in patients with immune checkpoint inhibitor-associated immune nephritis that are clonotypically identical to kidney T cell infiltrates. Oncoimmunology. (2022) 11:2124678. doi: 10.1080/2162402x.2022.2124678, PMID: 36185804 PMC9519023

[73] Gomez-Preciado F Martinez-Valenzuela L Anton-Pampols P Fulladosa X Tena MG Gomà M . Urinary soluble PD-1 as a biomarker of checkpoint inhibitor-induced acute tubulointerstitial nephritis. Clin Kidney J. (2024) 17:sfae200. doi: 10.1093/ckj/sfae200, PMID: 39131079 PMC11316395

[74] Isik B Alexander MP Manohar S Vaughan L KottsChade L Markovic S . Biomarkers, clinical features, and rechallenge for immune checkpoint inhibitor renal immune-related adverse events. Kidney Int Rep. (2021) 6:1022–31. doi: 10.1016/j.ekir.2021.01.013, PMID: 33912752 PMC8071627

[75] Seethapathy H Mistry K Sise ME . Immunological mechanisms underlying clinical phenotypes and noninvasive diagnosis of immune checkpoint inhibitor-induced kidney disease. Immunol Rev. (2023) 318:61–9. doi: 10.1111/imr.13243, PMID: 37482912 PMC10865966

[76] von Euw E Chodon T Attar N Jalil J Koya RC Comin-Anduix B . CTLA4 blockade increases Th17 cells in patients with metastatic melanoma. J Transl Med. (2009) 7:35. doi: 10.1186/1479-5876-7-35, PMID: 19457253 PMC2697137

[77] Bermejo S Bolufer M Riveiro-Barciela M Soler MJ . Immunotherapy and the spectrum of kidney disease: should we individualize the treatment? Front Med (Lausanne). (2022) 9:906565. doi: 10.3389/fmed.2022.906565, PMID: 35775000 PMC9237407

[78] Barbir EB Kitchlu A Herrmann SM . Immune checkpoint inhibitor-associated nephritis-treatment standard. Nephrol Dial Transplant. (2024) 39:1785–98. doi: 10.1093/ndt/gfae184, PMID: 39138117

[79] Ferreira CA Heidari P Ataeinia B Sinevici N Sise ME Colvin RB . Non-invasive detection of immunotherapy-induced adverse events. Clin Cancer Res. (2021) 27:5353–64. doi: 10.1158/1078-0432.Ccr-20-4641, PMID: 34253581 PMC8752648

[80] Gupta S Green-Lingren O Bhimaniya S Krokhmal A Jacene H Ostermann M . F18-FDG PET imaging as a diagnostic tool for immune checkpoint inhibitor-associated acute kidney injury. J Clin Invest. (2024) 134:e182275. doi: 10.1172/jci182275, PMID: 39115940 PMC11405038

[81] Seethapathy H Herrmann SM Rashidi A . Immune checkpoint inhibitor-associated AKI: debates in diagnosis, management, and rechallenge. Semin Nephrol. (2022) 42:151346. doi: 10.1016/j.semnephrol.2023.151346, PMID: 37137187

[82] Wang Y Xiong C Yu W Zhou M Shugg T Hsu FC . PCCA variant rs16957301 is a novel AKI risk genotype-specific for patients who receive ICI treatment: Real-world evidence from all of us cohort. Eur J Cancer. (2024) 213:115114. doi: 10.1016/j.ejca.2024.115114, PMID: 39536432 PMC11798912

[83] Monson KR Ferguson R Handzlik JE Xiong J Dagayev S Morales L . Tyrosine protein kinase SYK-related gene signature in baseline immune cells associated with adjuvant immunotherapy-induced immune-related adverse events in melanoma. Clin Cancer Res. (2024) 30:4412–23. doi: 10.1158/1078-0432.Ccr-24-0900, PMID: 39115425 PMC12269477

[84] Kobayashi M Numakura K Hatakeyama S Muto Y Sekine Y Sasagawa H . Severe immune-related adverse events in patients treated with nivolumab for metastatic renal cell carcinoma are associated with PDCD1 polymorphism. Genes (Basel). (2022) 13:1204. doi: 10.3390/genes13071204, PMID: 35885987 PMC9324515

[85] Lumlertgul N Vassallo P Tydeman F Lewis N Hobill A Weerapolchai K . Acute kidney injury in patients receiving immune checkpoint inhibitors: a retrospective real-world study. Eur J Cancer. (2023) 191:112967. doi: 10.1016/j.ejca.2023.112967, PMID: 37499561

[86] Abudayyeh A Suo L Lin H Mamlouk O Abdel-Wahab N Tchakarov A . Pathologic predictors of response to treatment of immune checkpoint inhibitor-induced kidney injury. Cancers (Basel). (2022) 14:5267. doi: 10.3390/cancers14215267, PMID: 36358686 PMC9656112

[87] Gupta S Garcia-Carro C Prosek JM Glezerman I Herrmann SM Garcia P . Shorter versus longer corticosteroid duration and recurrent immune checkpoint inhibitor-associated AKI. J Immunother Cancer. (2022) 10:e005646. doi: 10.1136/jitc-2022-005646, PMID: 36137651 PMC9511654

[88] Elghawy O Patel R Barsouk A Puthumana J Xu J Sussman J . Diagnosis, management, and outcomes of immune checkpoint inhibitor induced acute interstitial nephritis: A single-center experience. J Oncol Pharm Pract. (2025) 31:641–8. doi: 10.1177/10781552241252627, PMID: 38706192

[89] Zhou P Liu B Shen N Fan X Lu S Kong Z . Acute kidney injury in patients treated with immune checkpoint inhibitors: a single-center retrospective study. Ren Fail. (2024) 46:2326186. doi: 10.1080/0886022x.2024.2326186, PMID: 38466161 PMC10930152

[90] Lin JS Mamlouk O Selamet U Tchakarov A Glass WF Sheth RA . Infliximab for the treatment of patients with checkpoint inhibitor-associated acute tubular interstitial nephritis. Oncoimmunology. (2021) 10:1877415. doi: 10.1080/2162402x.2021.1877415, PMID: 33643693 PMC7872057

[91] Mamlouk O Lin JS Abdelrahim M Tchakarov AS Glass WF Selamet U . Checkpoint inhibitor-related renal vasculitis and use of rituximab. J Immunother Cancer. (2020) 8:e000750. doi: 10.1136/jitc-2020-000750, PMID: 32718987 PMC7380836

[92] Rassner M Baur R Wäsch R Schiffer M Schneider J Mackensen A . Two cases of carfilzomib-induced thrombotic microangiopathy successfully treated with Eculizumab in multiple myeloma. BMC Nephrol. (2021) 22:32. doi: 10.1186/s12882-020-02226-5, PMID: 33461512 PMC7814610

[93] Shivaraj K Tchakarov A Dong Y Lin JS . Tofacitinib for the treatment of refractory immune checkpoint inhibitor-associated immune nephritis. Clin Kidney J. (2024) 17:sfae127. doi: 10.1093/ckj/sfae127, PMID: 38803394 PMC11129582

[94] Herrmann SM Abudayyeh A Gupta S Gudsoorkar P Klomjit N Motwani SS . Diagnosis and management of immune checkpoint inhibitor-associated nephrotoxicity: a position statement from the American Society of Onco-nephrology. Kidney Int. (2025) 107:21–32. doi: 10.1016/j.kint.2024.09.017, PMID: 39455026

[95] Curkovic NB Irlmeier R Bai X Cui C Ye F Burnette HR . Impact of steroid dose and timing on efficacy of combination PD-1/CTLA-4 blockade. Oncoimmunology. (2025) 14:2494433. doi: 10.1080/2162402x.2025.2494433, PMID: 40248956 PMC12013437

[96] Luo J Beattie JA Fuentes P Rizvi H Egger JV Kern JA . Beyond steroids: immunosuppressants in steroid-refractory or resistant immune-related adverse events. J Thorac Oncol. (2021) 16:1759–64. doi: 10.1016/j.jtho.2021.06.024, PMID: 34265432 PMC8464489

[97] Barbir EB Mohan A Koirala P Gutierrez LE Giesen CD Lieske JC . Biomarker-guided infliximab therapy for immune checkpoint inhibitor-induced acute interstitial nephritis. Nephrol Dial Transplant. (2025) 40:602–4. doi: 10.1093/ndt/gfae257, PMID: 39516049

[98] Henderson Berg MH Del Rincón SV Miller WH . Potential therapies for immune-related adverse events associated with immune checkpoint inhibition: from monoclonal antibodies to kinase inhibition. J Immunother Cancer. (2022) 10:e003551. doi: 10.1136/jitc-2021-003551, PMID: 35086945 PMC8796266

[99] Zhao Q Zhang J Xu L Yang H Liang N Zhang L . Safety and efficacy of the rechallenge of immune checkpoint inhibitors after immune-related adverse events in patients with cancer: A systemic review and meta-analysis. Front Immunol. (2021) 12:730320. doi: 10.3389/fimmu.2021.730320, PMID: 34646270 PMC8503641

[100] Liu SJ Yan LJ Wang HC Ding ZN Liu H Zhang X . Safety, efficacy, and survival outcomes of immune checkpoint inhibitors rechallenge in patients with cancer: a systematic review and meta-analysis. Oncologist. (2024) 29:e1425–34. doi: 10.1093/oncolo/oyae134, PMID: 38940446 PMC11546642

[101] Xu S Shukuya T Tamura J Shimamura S Kurokawa K Miura K . Heterogeneous outcomes of immune checkpoint inhibitor rechallenge in patients with NSCLC: A systematic review and meta-analysis. JTO Clin Res Rep. (2022) 3:100309. doi: 10.1016/j.jtocrr.2022.100309, PMID: 35434666 PMC9011115

[102] Kitchlu A Jhaveri KD Sprangers B Yanagita M Wanchoo R . Immune checkpoint inhibitor use in patients with end-stage kidney disease: an analysis of reported cases and literature review. Clin Kidney J. (2021) 14:2012–22. doi: 10.1093/ckj/sfab090, PMID: 34476087 PMC8406068

[103] Hoffman-Censits J Pal S Kaiser C Ding B Bellmunt J . Atezolizumab in patients with renal insufficiency and mixed variant histology: analyses from an expanded access program in platinum-treated locally advanced or metastatic urothelial carcinoma. J Immunother Cancer. (2020) 8:e000419. doi: 10.1136/jitc-2019-000419, PMID: 32641319 PMC7342864

[104] Seydel F Delecluse S Zeier M Holland-Letz T Haag GM Berger AK . Efficacy and safety of checkpoint inhibitor treatment in patients with advanced renal or urothelial cell carcinoma and concomitant chronic kidney disease: A retrospective cohort study. Cancers (Basel). (2021) 13:1623. doi: 10.3390/cancers13071623, PMID: 33915693 PMC8036307

[105] Yun JW Kwon J Lim T . Long-term response of pembrolizumab in a patient with metastatic squamous non-small cell lung cancer on hemodialysis: case report and review of the literature. Med (Kaunas). (2023) 59:325. doi: 10.3390/medicina59020325, PMID: 36837526 PMC9967386

[106] Kobari Y Yoshida K Iizuka J Kondo T Ishida H Tanabe K . Three cases of nivolumab plus ipilimumab therapy in haemodialysis patients with metastatic renal cell carcinoma. In Vivo. (2021) 35:3585–9. doi: 10.21873/invivo.12663, PMID: 34697199 PMC8627745

[107] Sise ME Seethapathy H Reynolds KL . Diagnosis and management of immune checkpoint inhibitor-associated renal toxicity: illustrative case and review. Oncologist. (2019) 24:735–42. doi: 10.1634/theoncologist.2018-0764, PMID: 30902916 PMC6656503

[108] Engels EA Pfeiffer RM Fraumeni JF Jr. Kasiske BL Israni AK Snyder JJ . Spectrum of cancer risk among US solid organ transplant recipients. Jama. (2011) 306:1891–901. doi: 10.1001/jama.2011.1592, PMID: 22045767 PMC3310893

[109] Al-Adra D Al-Qaoud T Fowler K Wong G . De novo Malignancies after kidney transplantation. Clin J Am Soc Nephrol. (2022) 17:434–43. doi: 10.2215/cjn.14570920, PMID: 33782034 PMC8975024

[110] Nimmo A Elyan B Lakey J Marjoribanks S Methven S Morrison D . Increased cancer risk in kidney transplant patients in Scotland: a national registry linkage study. Br J Cancer. (2025) 133:555–63. doi: 10.1038/s41416-025-03086-2, PMID: 40517146 PMC12356882

[111] Yanik EL Clarke CA Snyder JJ Pfeiffer RM Engels EA . Variation in Cancer Incidence among Patients with ESRD during Kidney Function and Nonfunction Intervals. J Am Soc Nephrol. (2016) 27:1495–504. doi: 10.1681/asn.2015040373, PMID: 26563384 PMC4849829

[112] Leonforte F Mistretta A Nicosia V Micalizzi MC Londrigo D Giambra MM . Epidemiological overview of colorectal cancer in kidney transplant recipients: A systematic review. Cancers (Basel). (2025) 17:3352. doi: 10.3390/cancers17203352, PMID: 41154407 PMC12564217

[113] Blosser CD Haber G Engels EA . Changes in cancer incidence and outcomes among kidney transplant recipients in the United States over a thirty-year period. Kidney Int. (2021) 99:1430–8. doi: 10.1016/j.kint.2020.10.018, PMID: 33159960 PMC8096865

[114] Merzkani M Attieh RM Jhaveri KD Murakami N . Immunotherapy and cellular therapies for cancers in kidney transplant patients. Am J Nephrol. (2025), 1–14. doi: 10.1159/000544826, PMID: 39978323

[115] Legris T Sallée M Charmetant X Thaunat O Matignon M Joher N . Immune checkpoint inhibitors in kidney transplant recipients: A french multicenter retrospective cohort study. Transplant Direct. (2025) 11:e1851. doi: 10.1097/txd.0000000000001851, PMID: 40755508 PMC12316348

[116] Murakami N Mulvaney P Danesh M Abudayyeh A Diab A Abdel-Wahab N . A multi-center study on safety and efficacy of immune checkpoint inhibitors in cancer patients with kidney transplant. Kidney Int. (2021) 100:196–205. doi: 10.1016/j.kint.2020.12.015, PMID: 33359528 PMC8222056

[117] Abdel-Wahab N Safa H Abudayyeh A Johnson DH Trinh VA Zobniw CM . Checkpoint inhibitor therapy for cancer in solid organ transplantation recipients: an institutional experience and a systematic review of the literature. J Immunother Cancer. (2019) 7:106. doi: 10.1186/s40425-019-0585-1, PMID: 30992053 PMC6469201

[118] Kumar V Shinagare AB Rennke HG Ghai S Lorch JH Ott PA . The safety and efficacy of checkpoint inhibitors in transplant recipients: A case series and systematic review of literature. Oncologist. (2020) 25:505–14. doi: 10.1634/theoncologist.2019-0659, PMID: 32043699 PMC7288631

[119] Saleem N Wang J Rejuso A Teixeira-Pinto A Stephens JH Wilson A . Outcomes of solid organ transplant recipients with advanced cancers receiving immune checkpoint inhibitors: A systematic review and individual participant data meta-analysis. JAMA Oncol. (2025) 11:1150–9. doi: 10.1001/jamaoncol.2025.2374, PMID: 40545616 PMC12183638

[120] Khan MA Mehmood M El Azzazi H Shaikh S Bhasin-Chhabra B Gudsoorkar P . Immune checkpoint inhibitors and allograft rejection risk: emerging evidence regarding their use in kidney transplant recipients. J Clin Med. (2025) 14:5152. doi: 10.3390/jcm14145152, PMID: 40725844 PMC12295338

[121] Loupy A Certain A Tangprasertchai NS Racapé M Ursule-Dufait C Benbadi K . Evaluation of a decentralized donor-derived cell-free DNA assay for kidney allograft rejection monitoring. Transpl Int. (2024) 37:13919. doi: 10.3389/ti.2024.13919, PMID: 39741495 PMC11685011

[122] Rabant M Amrouche L Lebreton X Aulagnon F Benon A Sauvaget V . Urinary C-X-C motif chemokine 10 independently improves the noninvasive diagnosis of antibody-mediated kidney allograft rejection. J Am Soc Nephrol. (2015) 26:2840–51. doi: 10.1681/asn.2014080797, PMID: 25948873 PMC4625672

[123] Jacquemont L Tilly G Yap M Doan-Ngoc TM Danger R Guérif P . Terminally differentiated effector memory CD8(+) T cells identify kidney transplant recipients at high risk of graft failure. J Am Soc Nephrol. (2020) 31:876–91. doi: 10.1681/asn.2019080847, PMID: 32165419 PMC7191929

[124] Chesneau M Michel L Dugast E Chenouard A Baron D Pallier A . Tolerant kidney transplant patients produce B cells with regulatory properties. J Am Soc Nephrol. (2015) 26:2588–98. doi: 10.1681/asn.2014040404, PMID: 25644114 PMC4587683

[125] Tsung I Worden FP Fontana RJ . A pilot study of checkpoint inhibitors in solid organ transplant recipients with metastatic cutaneous squamous cell carcinoma. Oncologist. (2021) 26:133–8. doi: 10.1002/onco.13539, PMID: 32969143 PMC7873324

[126] Danesh MJ Mulvaney PM Murakami N Riella LV Silk AW Hanna GJ . Impact of corticosteroids on allograft protection in renal transplant patients receiving anti-PD-1 immunotherapy. Cancer Immunol Immunother. (2020) 69:1937–41. doi: 10.1007/s00262-020-02644-2, PMID: 32588077 PMC7479641

[127] Bolufer M Soler J Molina M Taco O Vila A Macía M . Immunotherapy for cancer in kidney transplant patients: A difficult balance between risks and benefits. Transpl Int. (2024) 37:13204. doi: 10.3389/ti.2024.13204, PMID: 39654653 PMC11625584

[128] Huang E Adjei M Peng A Najjar R Shoji J Chandran S . Tocilizumab treatment for microvascular inflammation and chronic active antibody-mediated rejection in kidney transplantation. Transplant Direct. (2025) 11:e1867. doi: 10.1097/txd.0000000000001867, PMID: 41058936 PMC12499727

[129] Noble J Comai G Corredetti V Laamech R Dard C Jouve T . Tocilizumab-based treatment of microvascular inflammation in kidney transplant recipients: A retrospective study. Transpl Int. (2025) 38:14502. doi: 10.3389/ti.2025.14502, PMID: 40454296 PMC12124137

[130] Moon S Jung M Go S Hong J Sohn HS Kim C . Engineered nanoparticles for enhanced antitumoral synergy between macrophages and T cells in the tumor microenvironment. Adv Mater. (2024) 36:e2410340. doi: 10.1002/adma.202410340, PMID: 39252658

[131] Liu P Guo J Xie Z Pan Y Wei B Peng Y . Co-delivery of aPD-L1 and CD73 inhibitor using calcium phosphate nanoparticles for enhanced melanoma immunotherapy with reduced toxicity. Adv Sci (Weinh). (2025) 12:e2410545. doi: 10.1002/advs.202410545, PMID: 39716993 PMC11831434

[132] Yu R Zhu B Chen D . Type I interferon-mediated tumor immunity and its role in immunotherapy. Cell Mol Life Sci. (2022) 79:191. doi: 10.1007/s00018-022-04219-z, PMID: 35292881 PMC8924142

[133] Hu M Zhou M Bao X Pan D Jiao M Liu X . ATM inhibition enhances cancer immunotherapy by promoting mtDNA leakage and cGAS/STING activation. J Clin Invest. (2021) 131:e139333. doi: 10.1172/jci139333, PMID: 33290271 PMC7843232

[134] Zhong N Zu Z Lu Y Sha X Li Y Liu Y . Mitochondria-targeted manganese-based mesoporous silica nanoplatforms trigger cGAS-STING activation and sensitize anti PD-L1 therapy in triple-negative breast cancer. Acta Biomater. (2025) 199:374–86. doi: 10.1016/j.actbio.2025.04.040, PMID: 40294811

[135] Li L Liu F Feng C Chen Z Zhang N Mao J . Role of mitochondrial dysfunction in kidney disease: Insights from the cGAS-STING signaling pathway. Chin Med J (Engl). (2024) 137:1044–53. doi: 10.1097/cm9.0000000000003022, PMID: 38445370 PMC11062705

[136] Zhu H Wang J Miao J Shen M Wang H Huang X . SNORD3A regulates STING transcription to promote ferroptosis in acute kidney injury. Adv Sci (Weinh). (2024) 11:e2400305. doi: 10.1002/advs.202400305, PMID: 38962954 PMC11434033

[137] Xu Z Yang X Lu X Su D Wang Y Wu H . PD-L1 antibody-modified plant-derived nanovesicles carrying a STING agonist for the combinational immunotherapy of melanoma. Biomaterials. (2025) 322:123396. doi: 10.1016/j.biomaterials.2025.123396, PMID: 40367814

[138] Yen YT Zhang Z Chen A Qiu Y Liu Q Wang Q . Enzymatically responsive nanocarriers targeting PD-1 and TGF-β pathways reverse immunotherapeutic resistance and elicit robust therapeutic efficacy. J Nanobiotechnol. (2025) 23:124. doi: 10.1186/s12951-025-03129-z, PMID: 39972327 PMC11841268

[139] Li Y Liu J Weichselbaum RR Lin W . Mitochondria-targeted multifunctional nanoparticles combine cuproptosis and programmed cell death-1 downregulation for cancer immunotherapy. Adv Sci (Weinh). (2024) 11:e2403520. doi: 10.1002/advs.202403520, PMID: 39013093 PMC11425249

[140] Rebeck ON Wallace MJ Prusa J Ning J Evbuomwan EM Rengarajan S . A yeast-based oral therapeutic delivers immune checkpoint inhibitors to reduce intestinal tumor burden. Cell Chem Biol. (2025) 32:98–110.e7. doi: 10.1016/j.chembiol.2024.10.013, PMID: 39571582 PMC11741927

[141] Kitchlu A Jhaveri KD Wadhwani S Deshpande P Harel Z Kishibe T . A systematic review of immune checkpoint inhibitor-associated glomerular disease. Kidney Int Rep. (2021) 6:66–77. doi: 10.1016/j.ekir.2020.10.002, PMID: 33426386 PMC7783581

